# Survival, treatment pattern, and treatment outcome in patients with cervical cancer metastatic to distant lymph nodes

**DOI:** 10.3389/fonc.2022.952480

**Published:** 2022-08-11

**Authors:** Haoliang Lin, Dongyan Wang, Hui Li, Chuling Wu, Fengqian Zhang, Zhongqiu Lin, Tingting Yao

**Affiliations:** ^1^ Department of Gynecological Oncology, Sun Yat-sen Memorial Hospital, Sun Yat-sen University, Guangzhou, China; ^2^ Guangdong Provincial Key Laboratory of Malignant Tumor Epigenetics and Gene Regulation, Sun Yat-sen Memorial Hospital, Sun Yat-sen University, Guangzhou, China

**Keywords:** cervical cancer, distant lymphatic metastasis, radiotherapy, brachytherapy, health services underutilization, treatment outcome, cause-specific survival

## Abstract

**Background:**

Cervical cancer with nodal involvement beyond the pelvis was considered as distant nodal metastasis in the previous International Federation of Gynecology and Obstetrics staging system. With the improvement of cancer-directed therapies, some of these patients can receive curative treatment. Classifying them as distant metastasis may result in underestimation of their prognosis as well as undertreatment. However, limited research has been conducted on the survival and treatment pattern in distant lymphatic metastatic cervical cancer.

**Objective:**

To investigate the survival, treatment pattern, and treatment outcome of patients with cervical cancer metastasized to distant lymph nodes (DLN) beyond the pelvis.

**Methods:**

Patients with stage III-IV cervical cancer from 1988 to 2016 were identified using the Surveillance, Epidemiology, and End Results program. The cancer cause-specific survival (CSS) was analyzed using the Kaplan-Meier method, log-rank test, multivariable Cox proportional hazard regression, subgroup analysis, and propensity score-matched analysis.

**Results:**

Of 17783 patients with stage III-IV cervical cancer, patients with distant nodal disease beyond the pelvis (n=1883; included para-aortic lymph nodes metastasis) had superior survival compared to those with pelvic organ invasion or with distant organ(s) metastasis (5-year CSS, 32.3%, 26.3%, and 11.5%, respectively; adjusted *P*<0.001). The T stage significantly affected the survival of patients with positive DLN (5-year CSS for T1, T2, and T3: 47.3%, 37.0%, and 19.8%, respectively, adjusted *P*<0.01). For patients with positive DLN, combination radiotherapy (external beam radiotherapy [EBRT] with brachytherapy) prolonged CSS compared to EBRT alone (5-year CSS, 38.0% vs 21.7%; propensity score-adjusted HR, 0.60; 95% CI 0.51-0.72; *P*<0.001). Despite the superiority of combination radiotherapy, EBRT was the most frequently used treatment after 2004 (483/1214, 39.8%), while the utilization of combination radiotherapy declined from 37.8% (253/669) during 1988 through 2003 to 25.2% (306/1214) during 2004 through 2016.

**Conclusion:**

Patients with cervical cancer metastasized to DLN have favorable survival compared to those with pelvic organ invasion or with distant organ(s) metastasis. Their prognosis is significantly affected by local tumor burden and local treatment. Adequate and aggressive local radiotherapy, such as image-guided brachytherapy, can be considered for these patients to achieve better outcomes.

## Introduction

Cervical cancer is the fourth most frequently diagnosed malignancy and the fourth leading cause of cancer death among females, causing an estimated 604,127 new cases and 341,831 deaths in 2020 worldwide (1). Historically, lymphatic spread beyond the pelvis was classified as distant metastasis and was staged as IVB in the previous International Federation of Gynecology and Obstetrics (FIGO) staging system for carcinoma of the cervix uteri (2). Some researchers had challenged this definition with the fact that patients with distant nodal metastasis lived much longer than those with bone or visceral organ disease (3–5). On the other hand, with the improvement of systemic therapy and radiotherapy, a part of patients with distant nodal metastasis (e.g., para-aortic lymph node metastasis, even supraclavicular lymph node involvement) can be treated by definitive chemoradiotherapy (6–8), suggesting that staging as IVB may lead to neglect and undertreatment of these patients. However, for patients with distant nodal disease, little published data summarized their prognostic performance and evaluate the quality of care in the real world. Given the paucity of data for this clinical situation, we used the Surveillance, Epidemiology, and End Results (SEER) Program to retrospectively investigate the clinicopathological features, survival, treatment pattern, and treatment outcome of patients with distant nodal metastasis.

## Materials and methods

### Study cohort, tumor staging, and clinical information

We used the SEER database to identify patients with newly diagnosed cervical cancer from 1988 through 2016. Providing information on cancer statistics of approximately 27.8% of the U.S. population, the SEER Program is publicly available and deidentified (9). Thus, this study was exempt from institutional review board approval. Patients were eligible if they had microscopically confirmed squamous cell carcinoma, adenocarcinoma, or adenosquamous carcinoma of cervix uteri and had a disease stage of III-IV according to the FIGO 2018 staging system. Patients with other histologic types, stage I-II disease, or incomplete TNM information to allow restaging were excluded. The study schema is shown in **Supplementary Figure S1**.

We defined distant lymph nodes (DLN) as lymph nodes beyond the pelvis, including para-aortic lymph nodes (PALN), mediastinal lymph nodes, supraclavicular lymph nodes, etc. We extracted eligible patients’ TNM stages and divided patients into six groups based on version 7 American Joint Committee on Cancer TNM staging for cervical cancer: T3aN0M0, T3bN0M0, T1-3N0M0, T1-3 with positive distant lymph nodes (DLN+), T4, and M1 group (**Supplementary Table S1**). M1 group includes patients with distant organ(s) metastasis with or without distant nodal involvement. From 1988 to 2003, PALN status was recorded separately, and we also reallocated patients to stage IIIA to IVB as stated in the FIGO 2018 staging system to examine its prognostic discriminatory ability.

All eligible patients’ demographic data, tumor characteristics, treatment, and survival outcomes were extracted. Local treatment indicated the performance of radiotherapy and surgery, including no radiotherapy or surgery, surgery only (hysterectomy or exenteration), radiotherapy only (combination radiotherapy [RT] or external beam radiotherapy [EBRT]), hysterectomy with postoperative radiotherapy (hysterectomy + combination RT or hysterectomy + EBRT), and other regimens. Combination RT was a treatment that united EBRT and brachytherapy. Pelvic lymphadenectomy performance was based on the SEER coding for “Regional Nodes” as in the prior study (10). Survival outcomes included cause of death and survival time after cervical cancer diagnosis.

### Statistical analysis

Baseline characteristics were compared using the chi-square test. Cause-specific survival (CSS) was chosen as the endpoint of our study, defined as the time interval from disease diagnosis to death from cancer of cervix uteri. The patients were censored from the time of last known follow-up or death of other cause. Our main objective was to examine survival of patients with distant nodal metastasis in a stage III to IV cohort. CSS was estimated by the Kaplan-Meier method and compared using the log-rank test. Multivariable Cox proportional hazard regression was adjusted for baseline factors to estimate hazard ratios (HRs). Variables with *P* value < 0.1 in the univariable analysis would enter the multivariable analysis.

Another purpose of our study was to identify prognostic factors and evaluate the efficacy of different treatment regimens in patients with distant nodal disease, using univariable survival analysis and multivariable Cox regression. Exploratory subgroup multivariable Cox regression analysis evaluating the impact of combination RT was performed. Propensity-score matched analysis (PSM) was performed comparing the oncologic outcome with combination RT versus EBRT. Multivariable logistic regression was performed to assess predictors for receiving combination RT versus EBRT. Significant predictors were used to generate the propensity scores. One-to-one nearest neighbor matching without replacement was performed to form the propensity-matched cohort, with caliper width of 0.15.

All *P* values were two-sided with the level of statistical significance set at < 0.05. The Benjamini-Hochberg procedure was used to control false discovery rate (FDR) for multiple comparisons, and the FDR adjusted *P* values < 0.05 were considered statistically significant (11, 12). All analyses were carried out using SPSS version 25 (IBM Corp. New York, U.S.A.).

## Results

### Patients characteristics

A total of 17763 patients at a median follow-up of 29.0 months met our inclusion criteria. Patient demographics and clinical characteristics are presented in **Table 1** and **Supplementary Table S2**. There were 1883 patients of the DLN+ group (**Table 2**), of which 517 patients were precisely staged as IIIC2. Patients with positive pelvic or distant lymph nodes tended to be younger, high-grade, with smaller primary tumor size, and more adenocarcinoma or adenosquamous carcinoma histology.

**Table 1:** Demographics and clinical characteristics of patients with stage III-IV cervical cancer.

	Total	T3aN0M0	T3bN0M0	T1-3N1M0	DLN+	T4	M1	*P* value
Characteristic	N=17763	n=739 (4.2%)	n=2761 (15.5%)	n=7318 (41.2%)	n=1883 (10.6%)	n=973 (5.5%)	n=4089 (23.0%)	
Age (years)								**<0.001**
<40	3641 (20.5%)	76 (10.3%)	355 (12.9%)	2215 (30.3%)	362 (19.2%)	104 (10.7%)	529 (12.9%)	
40-49	4517 (25.4%)	140 (18.9%)	632 (22.9%)	2135 (29.2%)	505 (26.8%)	191 (19.6%)	914 (22.4%)	
50-59	4249 (23.9%)	142 (19.2%)	744 (26.9%)	1585 (21.7%)	489 (26.0%)	260 (26.7%)	1029 (25.2%)	
60-69	2837 (16.0%)	144 (19.5%)	486 (17.6%)	847 (11.6%)	308 (16.4%)	200 (20.6%)	852 (20.8%)	
≥70	2519 (14.2%)	237 (32.1%)	544 (19.7%)	536 (7.3%)	219 (11.6%)	218 (22.4%)	765 (18.7%)	
**Race**								**<0.001**
White	13046 (73.4%)	497 (67.3%)	1934 (70.0%)	5501 (75.2%)	1397 (74.2%)	717 (73.7%)	3000 (73.4%)	
Black	2808 (15.8%)	154 (20.8%)	511 (18.5%)	987 (13.5%)	275 (14.6%)	174 (17.9%)	707 (17.3%)	
Others	1909 (10.7%)	88 (11.9%)	316 (11.4%)	830 (11.3%)	211 (11.2%)	82 (8.4%)	382 (9.3%)	
**Marital Status**								**<0.001**
Single	5133 (28.9%)	187 (25.3%)	810 (29.3%)	2146 (29.3%)	544 (28.9%)	275 (28.3%)	1171 (28.6%)	
Married	6971 (39.2%)	255 (34.5%)	911 (33.0%)	3269 (44.7%)	749 (39.8%)	296 (30.4%)	1491 (36.5%)	
Divorced	2333 (13.1%)	81 (11.0%)	396 (14.3%)	910 (12.4%)	250 (13.3%)	163 (16.8%)	533 (13.0%)	
Separated	441 (2.5%)	12 (1.6%)	84 (3.0%)	177 (2.4%)	45 (2.4%)	28 (2.9%)	95 (2.3%)	
Widowed	2202 (12.4%)	168 (22.7%)	471 (17.1%)	565 (7.7%)	217 (11.5%)	170 (17.5%)	611 (14.9%)	
Unknown	683 (3.8%)	36 (4.9%)	89 (3.2%)	251 (3.4%)	78 (4.1%)	41 (4.2%)	188 (4.6%)	
**Year of diagnosis**								**<0.001**
1988-1990	474 (2.7%)	16 (2.2%)	62 (2.2%)	195 (2.7%)	54 (2.9%)	19 (2.0%)	128 (3.1%)	
1991-1993	766 (4.3%)	36 (4.9%)	104 (3.8%)	322 (4.4%)	78 (4.1%)	25 (2.6%)	201 (4.9%)	
1994-1996	887 (5.0%)	46 (6.2%)	149 (5.4%)	344 (4.7%)	105 (5.6%)	40 (4.1%)	203 (5.0%)	
1997-1999	871 (4.9%)	51 (6.9%)	127 (4.6%)	329 (4.5%)	118 (6.3%)	29 (3.0%)	217 (5.3%)	
2000-2003	2958 (16.7%)	149 (20.2%)	558 (20.2%)	1045 (14.3%)	314 (16.7%)	166 (17.1%)	726 (17.8%)	
2004-2006	2377 (13.4%)	113 (15.3%)	461 (16.7%)	953 (13.0%)	241 (12.8%)	139 (14.3%)	470 (11.5%)	
2007-2009	2605 (14.7%)	108 (14.6%)	420 (15.2%)	1094 (14.9%)	265 (14.1%)	158 (16.2%)	560 (13.7%)	
2010-2012	2809 (15.8%)	97 (13.1%)	393 (14.2%)	1201 (16.4%)	302 (16.0%)	154 (15.8%)	662 (16.2%)	
2013-2016	4016 (22.6%)	123 (16.6%)	487 (17.6%)	1835 (25.1%)	406 (21.6%)	243 (25.0%)	922 (22.5%)	
**Histology**								**<0.001**
Squamous	14217 (80.0%)	629 (85.1%)	2456 (89.0%)	5833 (79.7%)	1520 (80.7%)	825 (84.8%)	2954 (72.2%)	
Adenocarcinoma	2621 (14.8%)	93 (12.6%)	237 (8.6%)	1035 (14.1%)	248 (13.2%)	112 (11.5%)	896 (21.9%)	
Adenosquamous	925 (5.2%)	17 (2.3%)	68 (2.5%)	450 (6.1%)	115 (6.1%)	36 (3.7%)	239 (5.8%)	
**Grade**								**<0.001**
G1	742 (4.2%)	42 (5.7%)	131 (4.7%)	303 (4.1%)	73 (3.9%)	57 (5.9%)	136 (3.3%)	
G2	5471 (30.8%)	233 (31.5%)	941 (34.1%)	2533 (34.6%)	503 (26.7%)	299 (30.7%)	962 (23.5%)	
G3	7379 (41.5%)	264 (35.7%)	935 (33.9%)	3133 (42.8%)	842 (44.7%)	366 (37.6%)	1839 (45.0%)	
Unknown	4171 (23.5%)	200 (27.1%)	754 (27.3%)	1349 (18.4%)	465 (24.7%)	251 (25.8%)	1152 (28.2%)	
**Tumor Size**								**<0.001**
≤4cm	3574 (20.1%)	113 (15.3%)	225 (8.1%)	2410 (32.9%)	333 (17.7%)	74 (7.6%)	419 (10.2%)	
>4cm	7746 (43.6%)	287 (38.8%)	1307 (47.3%)	3259 (44.5%)	921 (48.9%)	451 (46.4%)	1521 (37.2%)	
Unknown	6443 (36.3%)	339 (45.9%)	1229 (44.5%)	1649 (22.5%)	629 (33.4%)	448 (46.0%)	2149 (52.6%)	
Treatment^*^								**<0.001**
Untreated	1342 (7.6%)	57 (7.7%)	160 (5.8%)	150 (2.0%)	117 (6.2%)	110 (11.3%)	748 (18.3%)	
Treated	16421 (92.4%)	682 (92.3%)	2601 (94.2%)	7168 (98.0%)	1766 (93.8%)	863 (88.7%)	3341 (81.7%)	
**Surgery**								**<0.001**
No surgery	13249 (74.6%)	643 (87.0%)	2640 (95.6%)	4022 (55.0%)	1509 (80.1%)	836 (85.9%)	3599 (88.0%)	
Hysterectomy^†^	4381 (24.7%)	86 (11.6%)	105 (3.8%)	3273 (44.7%)	366 (19.4%)	87 (8.9%)	464 (11.3%)	
Exenteration^‡^	133 (0.7%)	10 (1.4%)	16 (0.6%)	23 (0.3%)	8 (0.4%)	50 (5.1%)	26 (0.6%)	
**Radiotherapy**								**<0.001**
No	3098 (17.4%)	88 (11.9%)	212 (7.7%)	786 (10.7%)	286 (15.2%)	178 (18.3%)	1548 (37.9%)	
Yes	14665 (82.6%)	651 (88.1%)	2549 (92.3%)	6532 (89.3%)	1597 (84.8%)	795 (81.7%)	2541 (62.1%)	
**Chemotherapy**								**<0.001**
No/Unknown	5191 (29.2%)	279 (37.8%)	757 (27.4%)	1820 (24.9%)	449 (23.8%)	321 (33.0%)	1565 (38.3%)	
Yes	12572 (70.8%)	460 (62.2%)	2004 (72.6%)	5498 (75.1%)	1434 (76.2%)	652 (67.0%)	2524 (61.7%)	

Number (%) is shown. Univariable analysis with chi-square test for *P* values. Significant *P* values are in bold form. ^*^Untreated: received no cancer-directed therapy; treated: received at least one kind of cancer-directed therapies. ^†^Hysterectomy includes total, modified radical or radical hysterectomy with or without removal of tubes and ovaries. ^‡^Exenteration includes anterior, posterior, total, or extended pelvic exenteration. DLN+, positive distant lymph nodes.

**Table 2:** Demographics and clinical characteristics of patients with distant lymph node metastasis.

Characteristic	T1-T2a	T2b-T3
	n=633 (33.6%)	n=1250 (66.4%)
**Age (years)**		
<40	154 (24.3%)	208 (16.6%)
40-49	197 (31.1%)	308 (24.6%)
50-59	147 (23.2%)	342 (27.4%)
60-69	80 (12.6%)	228 (18.2%)
≥70	55 (8.7%)	164 (13.1%)
**Race**		
White	472 (74.6%)	925 (74.0%)
Black	88 (13.9%)	187 (15.0%)
Others	73 (11.5%)	138 (11.0%)
**Year of diagnosis**		
1988-1990	21 (3.3%)	33 (2.6%)
1991-1993	27 (4.3%)	51 (4.1%)
1994-1996	33 (5.2%)	72 (5.8%)
1997-1999	50 (7.9%)	68 (5.4%)
2000-2003	104 (16.4%)	210 (16.8%)
2004-2006	72 (11.4%)	169 (13.5%)
2007-2009	97 (15.3%)	168 (13.4%)
2010-2012	106 (16.7%)	196 (15.7%)
2013-2016	123 (19.4%)	283 (22.6%)
**Marital Status**		
Single	183 (28.9%)	361 (28.9%)
Married	259 (40.9%)	490 (39.2%)
Divorced	90 (14.2%)	160 (12.8%)
Separated	21 (3.3%)	24 (1.9%)
Widowed	51 (8.1%)	166 (13.3%)
Unknown	29 (4.6%)	49 (3.9%)
**Histology**		
Squamous	467 (73.8%)	1053 (84.2%)
Adenocarcinoma	116 (18.3%)	132 (10.6%)
Adenosquamous	50 (7.9%)	65 (5.2%)
**Grade**		
G1	23 (3.6%)	50 (4.0%)
G2	174 (27.5%)	329 (26.3%)
G3	281 (44.4%)	561 (44.9%)
Unknown	155 (24.5%)	310 (24.8%)
**Tumor Size**		
≤4cm	190 (30.0%)	143 (11.4%)
>4cm	256 (40.4%)	665 (53.2%)
Unknown	187 (29.5%)	442 (35.4%)
**T stage**		
T1a/T2b	17 (2.7%)	433 (34.6%)
T1b/T3a	369 (58.3%)	159 (12.7%)
T1, NOS/T3b	63 (10.0%)	618 (49.4%)
T2a/T3, NOS	184 (29.1%)	40 (3.2%)
**N stage**		
N0	85 (13.4%)	146 (11.7%)
N1	475 (75.0%)	833 (66.6%)
NX	73 (11.5%)	271 (21.7%)
Radiation and surgery^*^		
No radiation or surgery	60 (9.5%)	155 (12.4%)
Surgery only	37 (5.8%)	34 (2.7%)
Combination RT^†^	164 (25.9%)	395 (31.6%)
EBRT	178 (28.1%)	475 (38.0%)
Hysterectomy + combination RT	42 (6.6%)	34 (2.7%)
Hysterectomy + EBRT	89 (14.1%)	64 (5.1%)
Other regimens^‡^	63 (10.0%)	93 (7.4%)
**Pelvic lymphadenectomy**		
No/Unknown	295 (46.6%)	866 (69.3%)
Yes	338 (53.4%)	384 (30.7%)
**Chemotherapy**		
No/Unknown	142 (22.4%)	307 (24.6%)
Yes	491 (77.6%)	943 (75.4%)

Number (%) is shown. ^*^Surgery includes hysterectomy and exenteration. Hysterectomy includes total, modified radical or radical hysterectomy with or without removal of tubes and ovaries. Exenteration includes anterior, posterior, total, or extended pelvic exenteration. ^†^Combination of EBRT and brachytherapy. ^‡^Other regimens include brachytherapy only, radiation (not otherwise specified, NOS) only, brachytherapy or radiation (not otherwise specified, NOS) after hysterectomy or exenteration, prior ± post-surgery radiotherapy, and intraoperative radiotherapy. EBRT, external beam radiotherapy; NOS, not otherwise specified; RT, radiotherapy.

### Treatment pattern

Patients with positive lymph nodes underwent more hysterectomy (T1-3N1M0, n=3273, 44.7%; DLN+, n=366, 19.4%). Among patients who were historically considered as stage IV diseases, those afflicted by pelvic organ invasion or distant organ(s) metastasis underwent less cancer-directed therapy (proportion of untreated patients: 6.2% for the DLN+ group, 11.3% for the T4 group, and 18.3% for the M1 group; adjusted *P*<0.001). More patients of the DLN+ group received radiotherapy (n=1597, 84.8%) and chemotherapy (n=1434, 76.5%) when compared to the T4 group and M1 group (adjusted *P*<0.05). The treatment pattern was similar in the 1988-2003 cohort (**Supplementary Table S2**).

**Figure 1:**
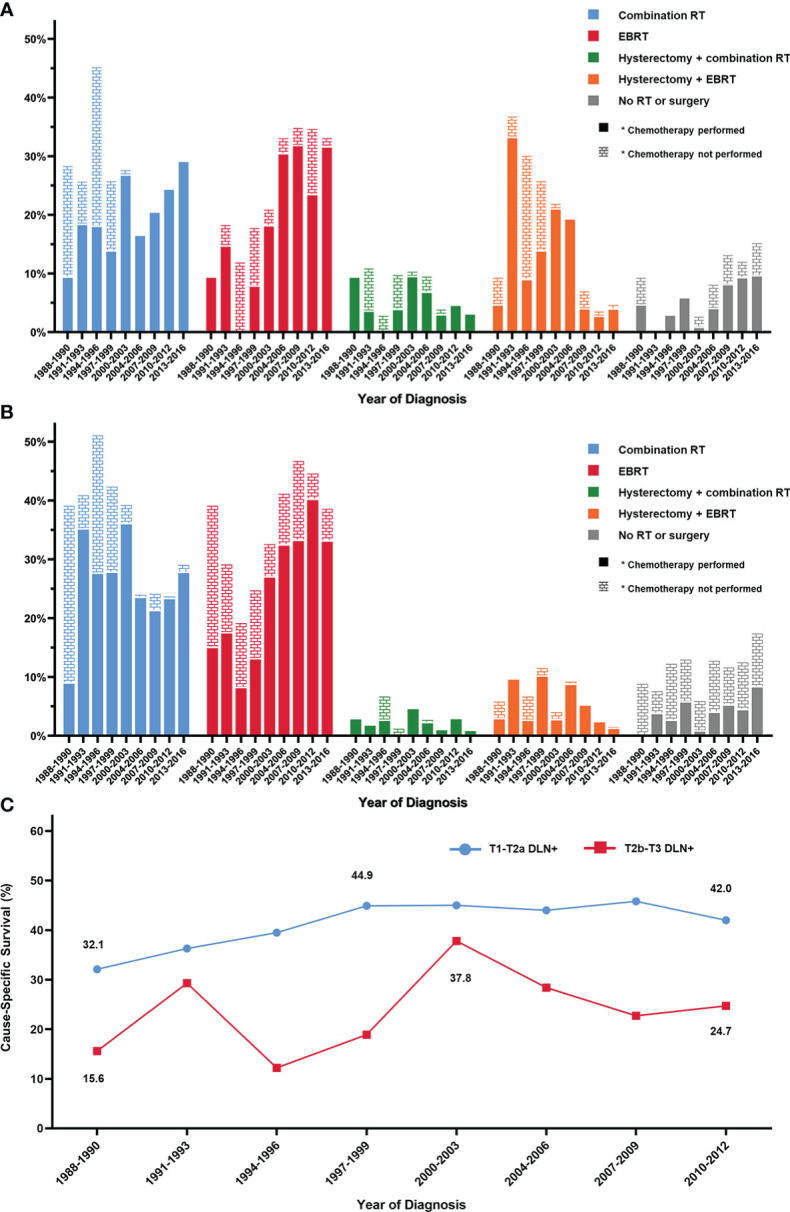
Changes of treatment pattern and survival for patients with distant lymph node metastasis. Treatment pattern for **(A)** patients with T1-T2a and distant lymph node metastasis, **(B)** patients with T2b-T3 and distant lymph node metastasis. **(C)** Changes of the 5-year cause-specific survival. EBRT, external beam radiotherapy; DLN+, positive distant lymph nodes; RT, radiotherapy.

We divided the DLN+ group into two subgroups by their T stage (**Table 2**) and summarized their treatment pattern in **Figure 1**. Six hundred and thirty-three patients (33.6%) had T1 and T2a stage diseases, while 1250 patients (66.4%) had T2b and T3 stage diseases. Radiation without surgery was the most common treatment regimen in both subgroups. Combination RT was the most frequently used local treatment during 1988 through 2003, received by 29.8% of patients with T1-T2a stage and 42.2% of patients with T2b-T3 stage. After 2004, the use of combination RT declined to 23.6% in the T1-T2a subgroup and 26.0% in the T2b-T3 subgroup, while EBRT became the most commonly used treatment.

For patients with T1-T2a stage, hysterectomy with postoperative radiotherapy (PORT) was received by 131 patients (20.7%), but this regimen gradually became infrequent from 2000 to 2016 (**Figure 1A**). From 1988 to 1999, 54.9% of patients from the DLN+ group received chemotherapy. After 2000, use of chemotherapy rapidly raised and fluctuated between 76.2% and 83.1%.

### Survival analysis

**Figure 2:**
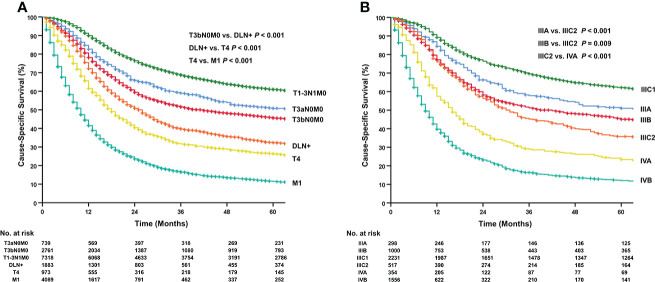
Kaplan-Meier curves for cause-specific survival. **(A)** stage III-IV cervical cancer (1988–2016). **(B)** stage III-IV cervical cancer (1988–2003). Trend analyses for all groups in two cohorts were significant. All *P* values had been adjusted by the Benjamini-Hochberg procedure and adjusted *P*<0.05 was considered statistically significant.

The 5-year CSS for T3aN0M0, T3bN0M0, T1-3N1M0, DLN+, T4, and M1 group was 50.8%, 45.6%, 61.0%, 32.3%, 26.3% and 11.5%, respectively (**Figure 2A**). For patients diagnosed between 1988 to 2003, the 5-year CSS for stage IIIA, IIIB, IIIC1, IIIC2, IVA and IVB was 51.0%, 45.1%, 62.2%, 35.8%, 23.4% and 12.0%, respectively (**Figure 2B**). All pairwise comparisons between two groups or stages were significant after Benjamini-Hochberg adjustment. Among patients of the DLN+ group, those with PALN metastasis lived longest (**Supplementary Figure S2**). The multivariable analysis also illustrated a favorable prognosis of the DLN+ group over the T4 and the M1 group (**Supplementary Table S3**). It was noteworthy that the revised FIGO stage was an independent prognostic factor for CSS (**Supplementary Table S3**). The survival of stage IIIC2 was superior to stage IVA but inferior to stage IIIB (IIIB: HR, 0.67; 95% CI, 0.58-0.77; *P*<0.001; IVA: HR, 1.38; 95% CI, 1.17-1.63; *P*<0.001; stage IIIC2 as reference).

**Figure 3:**
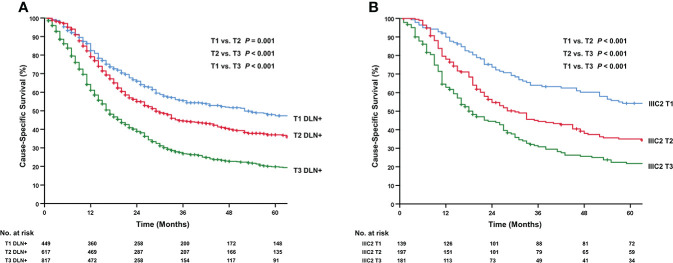
Kaplan-Meier curves for cervical cancer patients with distant lymph node metastasis based on different T stage. **(A)** DLN+ group (1988–2016). **(B)** stage IIIC2 (1988–2003). DLN+, positive distant lymph nodes. Trend analyses for two cohorts were significant. All *P* values had been adjusted by the Benjamini-Hochberg procedure and adjusted *P*<0.05 was considered statistically significant.

We compared CSS among patients in the DLN+ group based on T-stage and revealed significant differences. The 5-year CSS rate for T1, T2, and T3 was 47.3%, 37.0%, and 19.8%, respectively (adjusted *P*<0.01; **Figure 3A**). Likewise, heterogeneity of survival was found in stage IIIC2 (5-year CSS rate: 54.2% for T1, 35.0% for T2, 21.7% for T3, adjusted *P*<0.001; **Figure 3B**).

**Figure 4:**
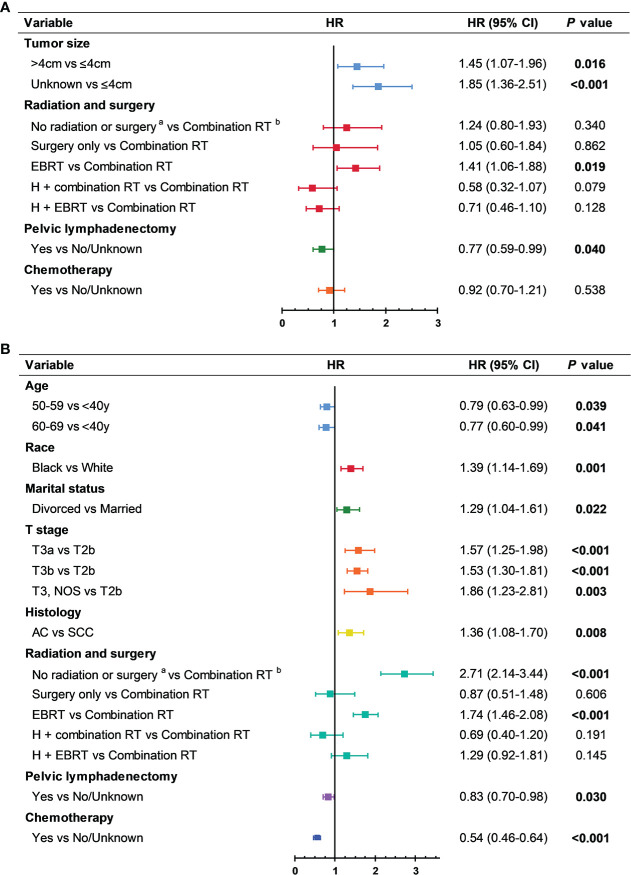
Prognostic factors of patients with distant lymph node metastasis. **(A)** T1-T2a and distant lymph node metastasis. Chemotherapy was forced into the multivariable model. **(B)** T2b-T3 and distant lymph node metastasis. AC, adenocarcinoma; CI, confidence interval; EBRT, external beam radiotherapy; H, hysterectomy; HR, multivariable adjusted hazard ratio; RT, radiotherapy; SCC, squamous cell carcinoma.

We performed multivariable analyses to identify predictors of CSS for patients in the DLN+ group. For the T1-T2a subgroup, significant predictors of CSS included tumor size and treatment regimens (**Figure 4A**, **Supplementary Table S4**). For the T2b-T3 subgroup, significant predictors of CSS included age, race, marital status, T stage, histology, and treatment regimens (**Figure 4B**, **Supplementary Table S5**).

Trends in 5-year CSS for patients with positive DLN are shown in **Figure 1C**. For the T1-T2a subgroup, the 5-year CSS generally increased from 32.1% during 1988 through 1990 to 44.9% during 1997 through 1999 but has since remained steady. For the T2b-T3 subgroup, the 5-year CSS has declined from a peak of 37.8% during 2000 through 2003 to 24.7% during 2010 through 2012.

### Treatment outcome

Treatment outcome for patients with positive DLN is summarized in **Figure 4**. The impact of chemotherapy on CSS differed across T stages. Chemotherapy reduced the risk of dying from cervical cancer by 46% in the T2b-T3 subgroup (HR, 0.54; 95% CI, 0.46-0.64; *P*<0.001). For the T1-T2a subgroup, this remarkable efficacy of chemotherapy was limited to patients received radiotherapy after 2000 (HR, 0.54; 95% CI, 0.33-0.90; *P*=0.017; **Supplementary Table S6**). In the T1-T2a subgroup, both PORT regimens were not associated with improved survival compared to combination RT after multivariable adjustment (hysterectomy + combination RT: HR, 0.58; 95% CI, 0.32-1.07; *P*=0.079; hysterectomy + EBRT: HR, 0.71; 95% CI, 0.46-1.10; *P*=0.128; combination RT as reference).

**Figure 5:**
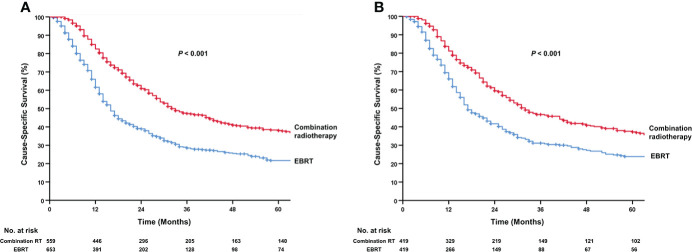
Kaplan-Meier curves for cause-specific survival in patients of DLN+ group treated by radiotherapy. **(A)** combination RT vs. EBRT. **(B)** combination RT vs. EBRT after propensity-score matched. DLN+, positive distant lymph nodes; EBRT, external beam radiotherapy; RT, radiotherapy.

Regarding radiotherapy, 559 patients of the DLN+ group received combination RT, and 653 patients received EBRT, without hysterectomy or exenteration. In multivariable logistic regression, age ≥ 60 years, black, and cases diagnosed after 2004 predicted decreased use of combination RT, whereas those who received pelvic lymphadenectomy and chemotherapy were more likely to undergo combination RT (**Supplementary Table S7**). Both univariable and multivariable analysis showed that combination RT can prolonged CSS compared with EBRT (5-year CSS, 38.0% vs 21.7%; median CSS, 33.0 vs 16.0 months; HR, 0.58; 95% CI 0.49-0.67; *P*<0.001; **Figure 5A**). The association of combination RT with prolonged CSS was maintained in the subgroup analysis for six covariates, including age, histology, T stage, year of diagnosis, pelvic lymphadenectomy, and chemotherapy performance (**Supplementary Figure 3**). PSM analysis was used to adjust for combination RT use. In the PSM cohort, combination RT group and EBRT group (419 patients for each group) were well-balanced (all predictors, standardized difference, <0.10 [**Supplementary Table S8**]); combination RT still improved CSS compared to EBRT (5-year CSS, 37.2% vs 23.9%; median CSS, 32.0 vs 17.0 months; HR, 0.60; 95% CI 0.51-0.72; *P*<0.001; **Figure 5B**).

## Discussion

Our study shows that patients with cervical cancer metastatic to distant lymph nodes presented a higher cause-specific survival than those with pelvic organ invasion or other distant metastases. The favorable survival of patients with positive DLN could be ascribed to their younger age, less impaired renal dysfunction caused by cervical cancer, and more receipt of cancer-directed therapies. Our study further addresses that the survival of patients with distant nodal metastasis varied widely by their local tumor burden. Patients with T1 and positive DLN had a comparable 5-year CSS to T3aN0M0 and T3bN0M0 (5-year CSS: 47.3% for T1 DLN+, 50.8% for T3aN0M0, and 45.6% for T3bN0M0), while the 5-year CSS of T3 and positive DLN was inferior to T4 (19.8% vs 26.3%).

Given the significant impact of local tumor burden, adequate treatment to primary cervix tumor is crucial even for patients with distant nodal disease. The association between improved local tumor control and prolonged survival had been illustrated by several articles regarding image-guided brachytherapy (IGBT) (13–15). For metastatic cervical cancer, increasing evidence had shown that local radiotherapy achieved better local control and more positively impacted patients’ survival than systemic chemotherapy alone (16–18). Among patients with distant nodal metastasis beyond the pelvis, definitive local radiotherapy is currently standard local treatment for those with positive PALN; and for patients with distant lymphatic spread beyond the abdomen, several retrospective studies with small sample sizes reported that local radiotherapy was a feasible method to control pelvic diseases and may achieve long-term survival (7, 19–23). Thus the National Comprehensive Cancer Network (NCCN) Guidelines suggest that patients with oligometastatic disease such as nodal metastases may benefit from aggressive local therapy, but this recommendation for local therapy is not clear (6). This population-based study further demonstrates that brachytherapy is an integral component of aggressive local radiation. Adding brachytherapy to the local radiation regimen for patients with distant lymphatic disease was associated with a 13% to 16% absolute decrement in death from cervical cancer at five years in comparison with EBRT alone. This result was also confirmed using subgroup analysis and PSM analysis.

Furthermore, our data pointed out an alarming real-world practice pattern for distant lymphatic metastatic cervical cancer. With the emerging use of intensity-modulated radiation therapy (IMRT) in the early 2000s (24, 25), EBRT became the mainstream local radiotherapy regimen in patients with positive DLN along with a worrisome underutilization of brachytherapy since 2004. Besides the impact of the technological advancement in EBRT, several possible causes associated with brachytherapy have driven physicians to omit combination RT, including increased treatment costs, high physician time requirements, low reimbursement, insufficient training during residency, and inadequate maintenance of brachytherapy skills (26–29). Consistent with the previous studies on locally advanced cervical carcinoma (26, 30), the growing trend away from brachytherapy was associated with a significant increase in mortality of patients with positive DLN in the era of modern conformal EBRT techniques. For example, survival of patients with T2b-T3 and positive DLN had improved during 2000 through 2003 in the wake of widespread chemotherapy uptake. After 2004, platinum-doublet regimens, which had a higher response rate over single-agent cisplatin, gradually became the most widely adopted systemic chemotherapy for metastatic diseases (31–33). However, the 5-year CSS of T2b-T3 and positive DLN, rather than picking up, rapidly declined from its peak of 37.8% to 24.7%, which coincided with the decreased brachytherapy utilization. Considering the limited benefit of platinum-doublet regimens in survival reported by trials (31–33), the progress in systemic chemotherapy was too modest to reverse the mortality impact of decreasing brachytherapy use. Fortunately, the EMBRACE research network, which focuses on IGBT in cervical cancer, retains a high utilization of brachytherapy in this scenario. Their further studies might optimize the chemoradiation strategies for distant lymphatic metastatic cervical cancer (34).

Hysterectomy is another effective method to eliminate early-stage cervical cancer but has limited practical application in distant nodal disease, which reflects in its decreasing use after 2000 (**Figure 1A**). The reduction in hysterectomy was largely due to the improved accuracy of preoperative imaging in detecting metastatic lymph nodes. In early-stage disease with suspicious lymph nodes on imaging, the current NCCN guideline prefers primary chemoradiation rather than hysterectomy with PORT, because multiple procedures are associated with increased morbidity (6). Our study illustrated that the survival benefit of completing hysterectomy was not significant when compared to combination RT. However, these data must be interpreted with caution because confounding by indication may exist in the analysis. Patients who received hysterectomy tended to have small metastatic nodes which were difficult to be detected on preoperative imaging, while those in whom hysterectomy was abandoned tended to have grossly enlarged nodes coupled with worse CSS. The SEER program did not record the reasons to complete or abandon hysterectomy and the timing of identifying positive nodes (e.g., preoperative, intraoperative, or postoperative). The unavailability of potential confounders hampers our elucidation on the actual effectiveness of hysterectomy in early-stage cervical cancer with distant lymph node metastasis. More well-designed studies are needed to reach a definitive conclusion on this topic.

Another significant result of this study is the interpretation of the 2018 FIGO staging system for cervical cancer. The 2018 FIGO staging system puts more emphasis on the distant nodal disease by reclassifying PALN metastasis as a new stage IIIC2 (35). Our results suggest that T1 and T2 PALN-positive patients had superior survival compared to those with stage IVA, while survival of T3 PALN-positive and stage IVA was similar, matching another population-based study (36). On the other hand, several studies have illustrated good oncologic outcomes with definitive concurrent chemoradiotherapy in treating PALN disease (37–40). Our results indicate that the disturbingly high rate of incomplete chemoradiation would lead to poorer survival in patients with distant nodal metastasis. Taken together, reclassifying PALN metastasis as stage IIIC2 not only improves prognostic discrimination of patients with distant metastasis but also assists physicians to determine the appropriate treatment. Notably, stage IIIC2 was a heterogeneous group of patients with various survival outcomes among T stage. The heterogeneity may lead to incorrect estimations of patients’ survival without consideration of the T stage. Besides, different proportions of T stage would result in various survival outcomes among cohorts, as an obstacle to comparing treatment’s efficacy among research.

This study has some limitations. First, the coding of metastatic lymph nodes location limits our analysis. In the SEER program, involvements of groin lymph nodes, mediastinal lymph nodes, and supraclavicular lymph nodes were all coded as “DLN metastasis”, except for PALN metastasis, which was recorded separately from 1988 to 2003. The rough coding system hindered survival estimation and assessment of treatment effectiveness for each lymph node group. Second, some treatment details were not available in the SEER program, including treatment intent, radiation dose, chemotherapy regimens, sequence of chemotherapy and radiation, use of bevacizumab, and types of lymphadenectomy. Third, exposure to brachytherapy may have been misclassified in a small number of patients (41). In our cohort, significant predictors for receiving combination RT were consistent with previous reports from the National Cancer Data Base (26, 42). Observed result of decreasing brachytherapy use in the early 2000s correlated with the increasing adoption of alternative radiotherapy such as IMRT. These facts reflect the high accuracy of radiotherapy coding in our cohort. Finally, potential bias may exist due to the unmeasured variables (e.g. performance status, comorbidity). To solve this issue, we used cancer-specific survival as the outcome, which depends less on patients’ health status. Several standard methods of limiting confounding were also performed, including multivariable analysis, subgroup analysis, and propensity score analysis.

## Conclusion

Patients with cervical cancer metastatic to DLN have favorable survival compared with those with pelvic organ invasion or with distant organ(s) metastasis. Given the significant impact of primary cervical tumor and the survival benefit from effective local treatment, patients afflicted by the distant nodal disease can be considered for more aggressive local treatment such as IGBT.

## Data availability statement

The raw data is publicly available on the SEER program (https://seer.cancer.gov). The processed data are available from the corresponding authors upon reasonable request.

## Ethics statement

Ethical review and approval was not required for the study on human participants in accordance with the local legislation and institutional requirements. Written informed consent for participation was not required for this study in accordance with the national legislation and the institutional requirements.

## Author contributions

Conceptualization: ZQL, TTY. Methodology: ZQL, TTY. Investigation: HLL, DYW. Formal analysis: HLL, DYW. Data Curation: HLL, DYW, HL. Validation: HL. Writing - Original Draft: HLL, DYW. Writing - Review and Editing: All authors. Visualization: HLL, CLW, FQZ. Supervision and Project administration: ZQL, TTY. Funding acquisition: TTY. All authors contributed to the article and approved the submitted version.

## Funding

This work was supported by National Natural Science Foundation of China (81572575), Guangdong province Natural Scientific Grant (2016A020215059&2021A1515010267), CSCO-Pilot Cancer Research Fund (Y-2019AZMS-0393).

## Acknowledgments

The authors wish to acknowledge the efforts of the SEER Program tumor registries in the creation of the SEER database. We are grateful to Ms. Wei-hua Xu for her advice and support on the statistics issue of this study. We also thank Dr. Hua-qiang Zhou’s support in accessing the SEER stat.

## Conflict of interest

The authors declare that the research was conducted in the absence of any commercial or financial relationships that could be construed as a potential conflict of interest.

## Publisher’s note

All claims expressed in this article are solely those of the authors and do not necessarily represent those of their affiliated organizations, or those of the publisher, the editors and the reviewers. Any product that may be evaluated in this article, or claim that may be made by its manufacturer, is not guaranteed or endorsed by the publisher.

## Supplementary material

Supplementary Figure 1 The overall flowchart. Patients were staged using the 2018 International Federation of Gynecology and Obstetrics staging system and the American Joint Committee on Cancer TNM 7^th^ edition staging system for cervical cancer. * Less common histologic types: including clear cell adenocarcinoma, small cell carcinoma, etc. AC, adenocarcinoma; ASC, adenosquamous carcinoma; DLN+, distant lymph node metastasis; SCC, squamous cell carcinoma.

Supplementary Figure 2 Kaplan-Meier curves for cause-specific survival in patients with distant lymph node metastasis (1988–2003). Distant lymph nodes include para-aortic lymph nodes (n=517) and other distant lymph nodes (ODLN, e.g. groin, mediastina, and supraclavicular lymph nodes; n=152). **(A)** IIIC2 vs. T1-3 ODLN+. **(B)** DLN+ vs. T4 (survival curves of stage IIIC2 and IVA as reference). DLN+, distant lymph node metastasis; ODLN+, other distant lymph node metastasis.

Supplementary Figure 3 Forest plot of the association between combination radiotherapy and cause-specific survival of patients with distant lymph node metastasis by subgroups. Combination RT: combination of EBRT and brachytherapy. CI, confidence interval; EBRT, external beam radiotherapy; HR, multivariable adjusted hazard ratio associated with combination RT (EBRT is the reference [HR, 1.0]); RT, radiotherapy

Supplementary Table 1 The relationship between patients’ groups and the 2018 FIGO staging system

Supplementary Table 2 Demographics and clinical characteristics of patients with stage III-IV cervical cancer diagnosed from 1988 to 2003

Supplementary Table 3 Multivariable analysis for cause-specific survival of patients with stage III-IV cervical cancer

Supplementary Table 4 Univariable and multivariable analysis for cause-specific survival of patients with T1-T2a and distant lymph node metastasis

Supplementary Table 5 Univariable and multivariable analysis for cause-specific survival of patients with T2b-T3 and distant lymph node metastasis

Supplementary Table 6 Subgroup analysis of the association between chemotherapy and cause-specific survival of patients with T1-T2a and distant lymph node metastasis.

Supplementary Table 7 Demographics and clinical characteristics of patients receiving combination radiotherapy and external beam radiotherapy

Supplementary Table 8 Demographics and clinical characteristics of patients receiving combination radiotherapy and external beam radiotherapy for the propensity–score matched analysis

Supplementary Table S9 Results of previous studies of chemoradiotherapy of patients with metastatic cervical cancer.
