# Supplementary figures and images for: Survival, treatment pattern, and treatment outcome in patients with cervical cancer metastatic to distant lymph nodes
Source: Front Oncol. 2022 Aug 11;12:952480. doi: 10.3389/fonc.2022.952480 (PMC9402899; doi:10.3389/fonc.2022.952480)

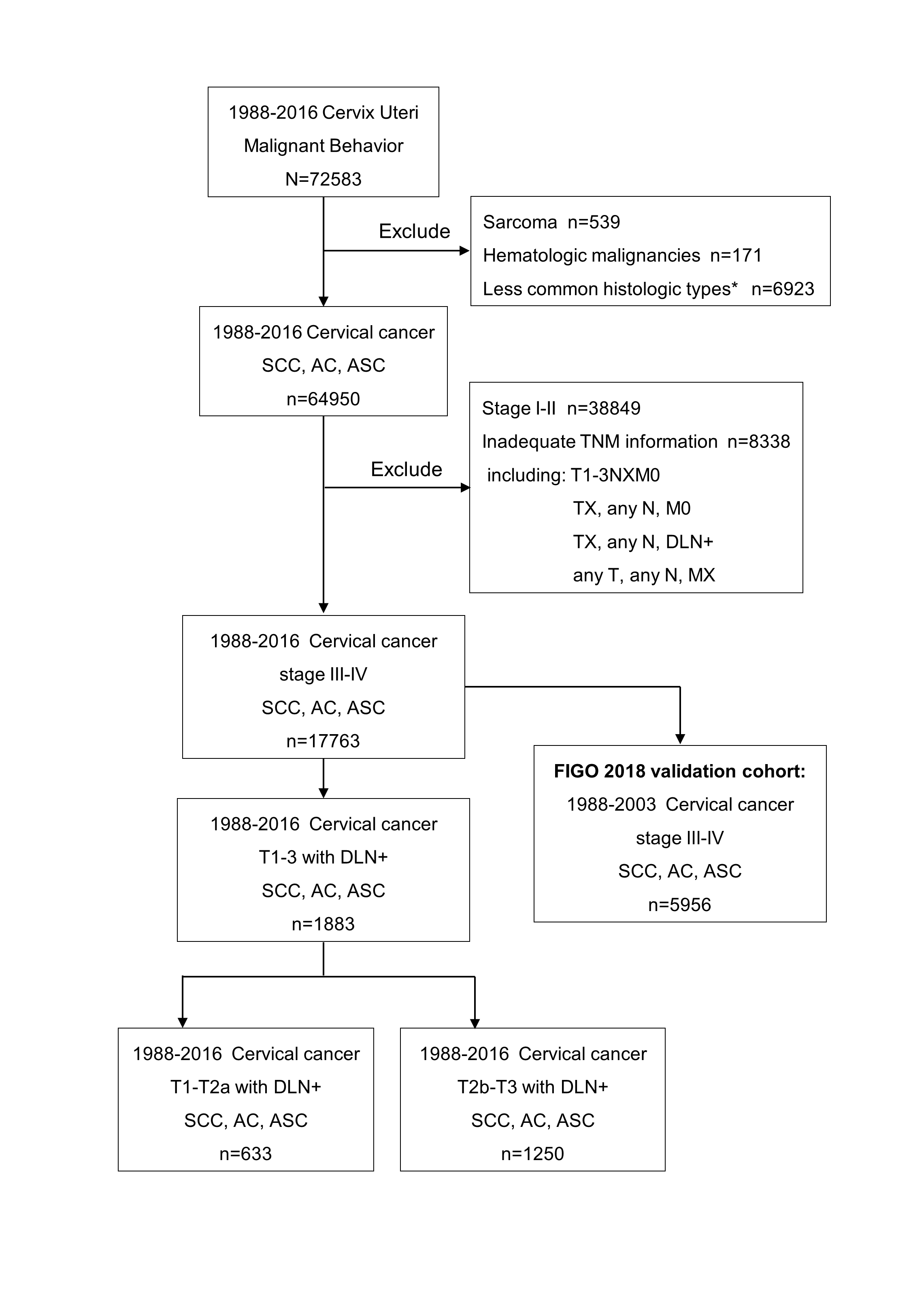

Supplement: Supplementary Figure 1 — The overall flowchart. Patients were staged using the 2018 International Federation of Gynecology and Obstetrics staging system and the American Joint Committee on Cancer TNM 7th edition staging system for cervical cancer. * Less common histologic types: including clear cell adenocarcinoma, small cell carcinoma, etc. AC, adenocarcinoma; ASC, adenosquamous carcinoma; DLN+, distant lymph node metastasis; SCC, squamous cell carcinoma. [file Image_1.tif]

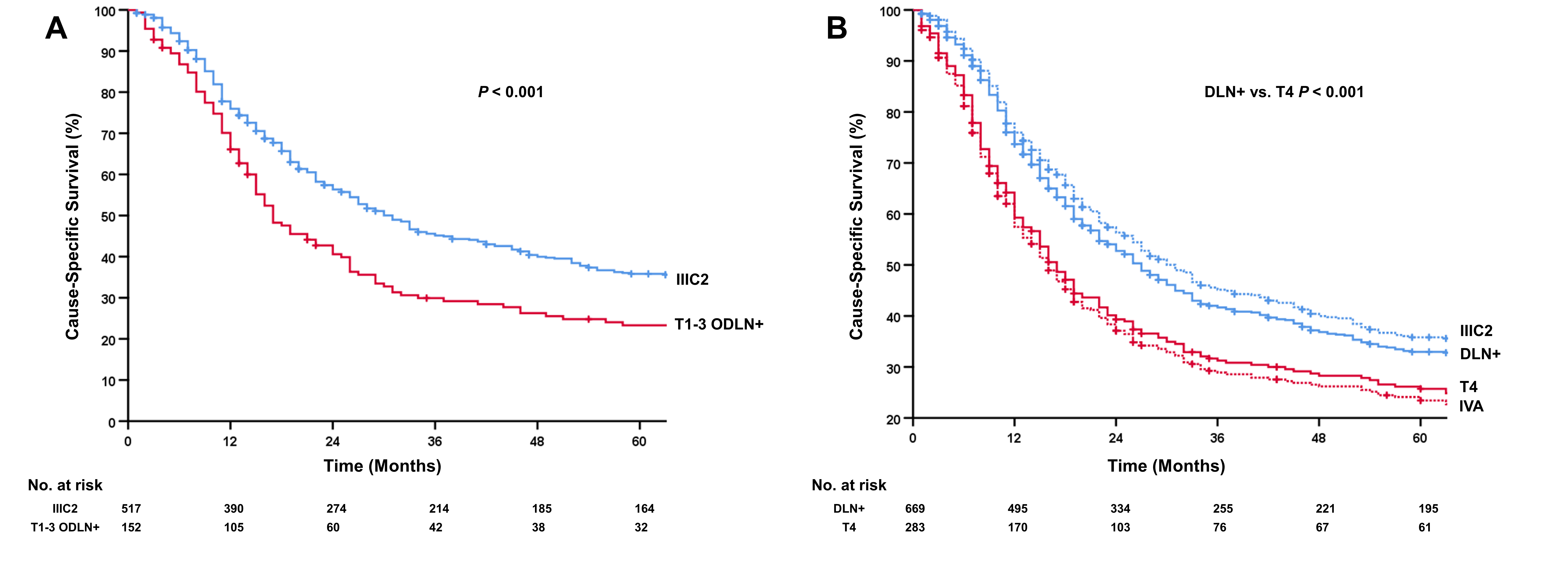

Supplement: Supplementary Figure 2 — Kaplan-Meier curves for cause-specific survival in patients with distant lymph node metastasis (1988–2003). Distant lymph nodes include para-aortic lymph nodes (n=517) and other distant lymph nodes (ODLN, e.g. groin, mediastina, and supraclavicular lymph nodes; n=152). (A) IIIC2 vs. T1-3 ODLN+. (B) DLN+ vs. T4 (survival curves of stage IIIC2 and IVA as reference). DLN+, distant lymph node metastasis; ODLN+, other distant lymph node metastasis. [file Image_2.tif]

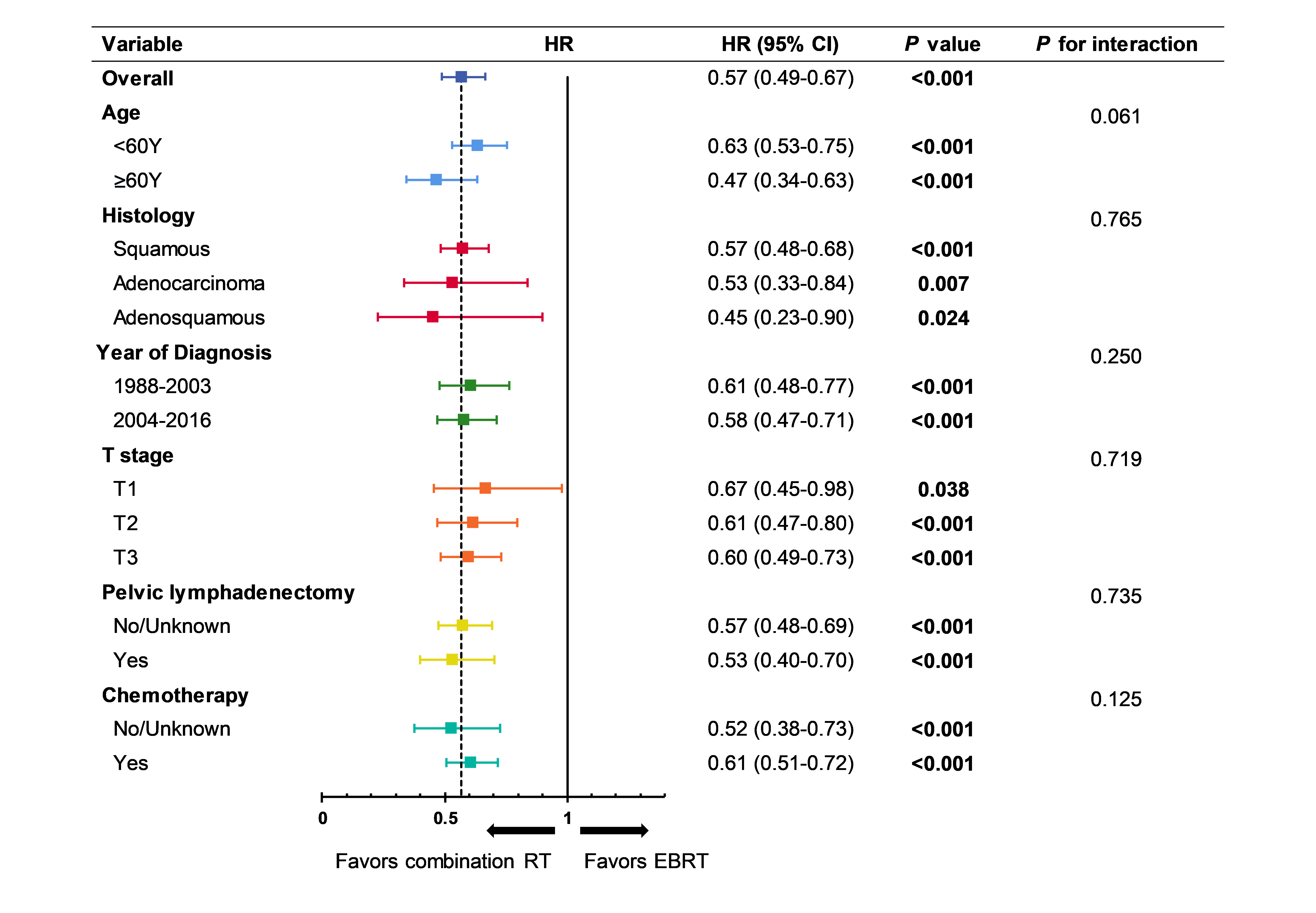

Supplement: Supplementary Figure 3 — Forest plot of the association between combination radiotherapy and cause-specific survival of patients with distant lymph node metastasis by subgroups. Combination RT: combination of EBRT and brachytherapy. CI, confidence interval; EBRT, external beam radiotherapy; HR, multivariable adjusted hazard ratio associated with combination RT (EBRT is the reference [HR, 1.0]); RT, radiotherapy [file Image_3.tif]
