# Supplementary material for: Survival, treatment pattern, and treatment outcome in patients with cervical cancer metastatic to distant lymph nodes
Source: Front Oncol. 2022 Aug 11;12:952480. doi: 10.3389/fonc.2022.952480 (PMC9402899; doi:10.3389/fonc.2022.952480)
Supplement: Supplementary Table 1 — The relationship between patients’ groups and the 2018 FIGO staging system [file DataSheet_1.docx]

Title: Survival, treatment pattern, and treatment outcome in patients with cervical cancer metastatic to distant lymph nodes

Supplementary Tables

| **Supplementary Table 1. The relationship between patients’ groups and the 2018 FIGO staging system** | | | | |
| --- | --- | --- | --- | --- |
| **When T is** | **And N is** | **And M is** | **Then the group** * **is** | **the FIGO stage** ^†^ **is** |
| T3a | N0 | M0 | T3aN0M0 | IIIA |
| T3b | N0 | M0 | T3bN0M0 | IIIB |
| T1-3 | N1 | M0 | T1-3N1M0 | IIIC1 |
| T1-3 | Any N | PALN+ only ^‡^ | DLN+ | IIIC2 |
| T1-3 | Any N | ODLN+ only ^§^ | DLN+ | IVB |
| T4 | Any N | M0 | T4 | IVA |
| T4 | Any N | PALN+ only | M1 | IVA |
| Any T | Any N | Distant organ+ with or without DLN+ ^‖^ | M1 | IVB |
| 2018 FIGO and AJCC Tumor-Node-Metastases (TNM) 7^th^ edition staging systems for stage III-IV carcinoma of the uterine cervix were used. Distant lymph nodes include para-aortic lymph nodes, groin lymph nodes, mediastinal lymph nodes, and supraclavicular lymph nodes. ^*^ Groups for 1988-2016 cohort. ^†^ Tumor staging for 1988-2003 cohort. ^‡^ PALN+ only: para-aortic lymph node metastasis. ^§^ ODLN+ only: other distant lymph node metastases, including metastases to groin lymph nodes, mediastinal lymph nodes, and supraclavicular lymph nodes. ^‖^ Distant organ(s) metastasis with or without distant lymph node metastasis. Abbreviations: AJCC, American Joint Committee on Cancer; DLN, distant lymph nodes; FIGO, International Federation of Gynecology and Obstetrics; ODLN, other distant lymph nodes; PALN, para-aortic lymph nodes | | | | |

| **Supplementary Table 2. Demographics and clinical characteristics of patients with stage III-IV cervical cancer diagnosed from 1988 to 2003** | | | | | | | | |
| --- | --- | --- | --- | --- | --- | --- | --- | --- |
|  | **Total** | **Stage IIIA** | **Stage IIIB** | **Stage IIIC1** | **Stage IIIC2** | **Stage IVA** | **Stage IVB** | ***P* value** |
| **Characteristic** | **N=5956** | **n=298 (5.0%)** | **n=1000 (16.8%)** | **n=2231 (37.5%)** | **n=517 (8.7%)** | **n=354 (5.9%)** | **n=1556 (26.1%)** |  |
| **Age (years)** |  |  |  |  |  |  |  | **<0.001** |
| <40 | 1302 (21.9%) | 35 (11.7%) | 145 (14.5%) | 764 (14.5%) | 115 (22.2%) | 45 (12.7%) | 198 (12.7%) |  |
| 40-49 | 1596 (26.8%) | 54 (18.1%) | 225 (22.5%) | 717 (32.1%) | 163 (31.5%) | 72 (20.3%) | 365 (23.5%) |  |
| 50-59 | 1255 (21.1%) | 54 (18.1%) | 248 (24.8%) | 409 (18.3%) | 122 (23.6%) | 92 (26.0%) | 330 (21.2%) |  |
| 60-69 | 883 (14.8%) | 56 (18.8%) | 172 (17.2%) | 208 (9.3%) | 72 (13.9%) | 67 (18.9%) | 308 (19.8) |  |
| ≥70 | 920 (15.4%) | 99 (33.2%) | 210 (21.0%) | 133 (6.0%) | 45 (8.7%) | 78 (22.0%) | 355 (22.8%) |  |
| **Race** |  |  |  |  |  |  |  | **<0.001** |
| White | 4398 (73.8%) | 197 (66.1%) | 704 (70.4%) | 1694 (75.9%) | 388 (75.0%) | 263 (74.3%) | 1152 (74.0%) |  |
| Black | 956 (16.1%) | 68 (22.8%) | 193 (19.3%) | 296 (13.3%) | 67 (13.0%) | 59 (16.7%) | 273 (17.5%) |  |
| Others | 602 (10.1%) | 33 (11.1%) | 103 (10.3%) | 241 (10.8%) | 62 (12.0%) | 32 (9.0%) | 131 (8.4%) |  |
| **Marital Status** |  |  |  |  |  |  |  | **<0.001** |
| Single | 1404 (23.6%) | 76 (25.5%) | 233 (23.3%) | 536 (24.0%) | 117 (22.6%) | 76 (21.5%) | 366 (23.5%) |  |
| Married | 2487 (41.8%) | 100 (33.6%) | 357 (35.7%) | 1083 (48.5%) | 242 (46.8%) | 125 (35.3%) | 580 (37.3%) |  |
| Divorced | 801 (13.4%) | 28 (9.4%) | 142 (14.2%) | 310 (13.9%) | 70 (13.5%) | 56 (15.8%) | 195 (12.5%) |  |
| Separated | 153 (2.6%) | 6 (2.0%) | 37 (3.7%) | 46 (2.1%) | 10 (1.9%) | 11 (3.1%) | 43 (2.8%) |  |
| Widowed | 925 (15.5%) | 78 (26.2%) | 198 (19.8%) | 199 (8.9%) | 64 (12.4%) | 72 (20.3%) | 314 (20.2%) |  |
| Unknown | 186 (3.1%) | 10 (3.4%) | 33 (3.3%) | 57 (2.6%) | 14 (2.7%) | 14 (4.0%) | 58 (3.7%) |  |
| **Year of diagnosis** |  |  |  |  |  |  |  | **<0.001** |
| 1988-1990 | 474 (8.0%) | 16 (5.4%) | 62 (6.2%) | 195 (8.7%) | 40 (7.7%) | 21 (5.9%) | 140 (9.0%) |  |
| 1991-1993 | 766 (12.9%) | 36 (12.1%) | 104 (10.4%) | 322 (14.4%) | 67 (13.0%) | 34 (9.6%) | 203 (13.0%) |  |
| 1994-1996 | 887 (14.9%) | 46 (15.4%) | 149 (14.9%) | 344 (15.4%) | 90 (17.4%) | 53 (15.0%) | 205 (13.2%) |  |
| 1997-1999 | 871 (14.6%) | 51 (17.1%) | 127 (12.7%) | 329 (14.7%) | 83 (16.1%) | 43 (12.1%) | 238 (15.3%) |  |
| 2000-2003 | 2958 (49.7%) | 149 (50.0%) | 558 (55.8%) | 1041 (46.7%) | 237 (45.8%) | 203 (57.3%) | 770 (49.5%) |  |
| **Histology** |  |  |  |  |  |  |  | **<0.001** |
| Squamous | 4748 (79.7%) | 258 (86.6%) | 888 (88.8%) | 1768 (79.2%) | 413 (79.9%) | 291 (82.2%) | 1130 (72.6%) |  |
| Adenocarcinoma | 804 (13.5%) | 30 (10.1%) | 77 (7.7%) | 280 (12.6%) | 67 (13.0%) | 44 (12.4%) | 306 (19.7%) |  |
| Adenosquamous | 404 (6.8%) | 10 (3.4%) | 35 (3.5%) | 183 (8.2%) | 37 (7.2%) | 19 (5.4%) | 120 (7.7%) |  |
| **Grade** |  |  |  |  |  |  |  | **<0.001** |
| G1 | 266 (4.5%) | 21 (7.0%) | 58 (5.8%) | 92 (4.1%) | 16 (3.1%) | 25 (7.1%) | 54 (3.5%) |  |
| G2 | 1748 (29.3%) | 94 (31.5%) | 306 (30.6%) | 769 (34.5%) | 145 (28.0%) | 103 (29.1%) | 331 (21.3%) |  |
| G3 | 2626 (44.1%) | 102 (34.2%) | 356 (35.6%) | 1014 (45.5%) | 246 (47.6%) | 147 (41.5%) | 761 (48.9%) |  |
| Unknown | 1316 (22.1%) | 81 (27.2%) | 280 (28.0%) | 356 (16.0%) | 110 (21.3%) | 79 (22.3%) | 410 (26.3%) |  |
| **Tumor Size** |  |  |  |  |  |  |  | 0.547 |
| ≤4cm | 1202 (20.2%) | 60 (20.1%) | 207 (20.7%) | 454 (20.3%) | 106 (20.5%) | 65 (18.4%) | 310 (19.9%) |  |
| >4cm | 1970 (33.1%) | 103 (34.6%) | 348 (34.8%) | 706 (31.6%) | 166 (32.1%) | 133 (37.6%) | 514 (33.0%) |  |
| Unknown | 2784 (46.7%) | 135 (45.3%) | 445 (44.5%) | 1071 (48.0%) | 245 (47.4%) | 156 (44.1%) | 732 (47.0%) |  |
| **Treatment ^*^** |  |  |  |  |  |  |  | **<0.001** |
| Untreated | 424 (7.1%) | 14 (4.7%) | 48 (4.8%) | 40 (1.8%) | 19 (3.7%) | 34 (9.6%) | 269 (17.3%) |  |
| Treated | 5532 (92.9%) | 284 (95.3%) | 952 (95.2%) | 2191 (98.2%) | 498 (96.3%) | 320 (90.4%) | 1287 (82.7%) |  |
| **Surgery** |  |  |  |  |  |  |  | **<0.001** |
| No surgery | 3992 (67.0%) | 246 (82.6%) | 953 (95.3%) | 824 (36.9%) | 349 (67.5%) | 276 (78.0%) | 1344 (86.4%) |  |
| Hysterectomy ^†^ | 1898 (31.9%) | 45 (15.1%) | 43 (4.3%) | 1394 (62.5%) | 165 (31.9%) | 51 (14.4%) | 200 (12.9%) |  |
| Exenteration ^‡^ | 66 (1.1%) | 7 (2.3%) | 4 (0.4%) | 13 (0.6%) | 3 (0.6%) | 27 (7.6%) | 12 (0.8%) |  |
| **Radiotherapy** |  |  |  |  |  |  |  | **<0.001** |
| No | 1054 (17.7%) | 25 (8.4%) | 62 (6.2%) | 314 (14.1%) | 50 (9.7%) | 62 (17.5%) | 541 (34.8%) |  |
| Yes | 4902 (82.3%) | 273 (91.6%) | 938 (93.8%) | 1917 (85.9%) | 467 (90.3%) | 292 (82.5%) | 1015 (65.2%) |  |
| **Chemotherapy** |  |  |  |  |  |  |  | **<0.001** |
| No/ Unknown | 2601 (43.7%) | 150 (50.3%) | 386 (38.6%) | 1042 (46.7%) | 168 (32.5%) | 142 (40.1%) | 713 (45.8%) |  |
| Yes | 3355 (56.3%) | 148 (49.7%) | 614 (61.4%) | 1189 (53.3%) | 349 (67.5%) | 212 (59.9%) | 843 (54.2%) |  |
| Number (%) is shown. Univariable analysis with chi-square test for *P* values. Significant *P* values are in bold form. ^*^ Untreated: received no cancer-directed therapy; treated: received at least one kind of cancer-directed therapies. ^†^ Hysterectomy includes total, modified radical or radical hysterectomy with or without removal of tubes and ovaries. ^‡^ Exenteration includes anterior, posterior, total, or extended pelvic exenteration. | | | | | | | | |

| **Supplementary Table 3. Multivariable analysis for cause-specific survival of patients with stage III-IV cervical cancer** | | | | |
| --- | --- | --- | --- | --- |
|  | **1988-2003** | | **1988-2016** | |
| **Characteristic** | **HR (95%CI)** | ***P*** | **HR (95%CI)** | ***P*** |
| **Age (years)** |  |  |  |  |
| <40 | Ref | - | Ref | - |
| 40-49 | 1.01 (0.91-1.12) | 0.845 | 1.01 (0.95-1.08) | 0.715 |
| 50-59 | 1.11 (0.99-1.23) | 0.065 | 1.04 (0.97-1.11) | 0.280 |
| 60-69 | 1.09 (0.97-1.23) | 0.136 | 1.06 (0.99-1.15) | 0.102 |
| ≥70 | 1.39 (1.24-1.57) | **<0.001** | 1.33 (1.23-1.45) | **<0.001** |
| **Race** |  |  |  |  |
| White | Ref | - | Ref | - |
| Black | 1.22 (1.11-1.33) | **<0.001** | 1.18 (1.12-1.25) | **<0.001** |
| Others | 0.87 (0.77-0.98) | **0.020** | 0.91 (0.85-0.98) | **0.008** |
| **Marital Status** |  |  |  |  |
| Single | - | - | 1.11 (1.06-1.17) | **<0.001** |
| Married | - | - | Ref | - |
| Divorced | - | - | 1.15 (1.08-1.23) | **<0.001** |
| Separated | - | - | 1.13 (0.98-1.29) | 0.090 |
| Widowed | - | - | 1.09 (1.01-1.17) | **0.019** |
| Unknown | - | - | 1.01 (0.90-1.12) | 0.913 |
| **Year of diagnosis** |  |  |  |  |
| 1988-1990 | - | - | - | - |
| 1991-1993 | - | - | - | - |
| 1994-1996 | - | - | - | - |
| 1997-1999 | - | - | - | - |
| 2000-2003 | - | - | - | - |
| 2004-2006 | - | - | - | - |
| 2007-2009 | - | - | - | - |
| 2010-2012 | - | - | - | - |
| 2013-2016 | - | - | - | - |
| **Stage/ Group** |  |  |  |  |
| IIIA / T3aN0M0 | 0.57 (0.47-0.70) | **<0.001** | 0.51 (0.45-0.58) | **<0.001** |
| IIIB / T3bN0M0 | 0.67 (0.58-0.77) | **<0.001** | 0.63 (0.58-0.69) | **<0.001** |
| IIIC1 / T1-3N1M0 | 0.61 (0.53-0.69) | **<0.001** | 0.53 (0.48-0.57) | **<0.001** |
| IIIC2 / DLN+ | Ref | - | Ref | - |
| IVA / T4 | 1.38 (1.17-1.63) | **<0.001** | 1.14 (1.04-1.26) | **0.008** |
| IVB / Other M1 | 1.96 (1.73-2.23) | **<0.001** | 1.91 (1.79-2.05) | **<0.001** |
| **Histology** |  |  |  |  |
| Squamous | Ref |  | Ref | - |
| Adenocarcinoma | 1.23 (1.12-1.36) | **<0.001** | 1.14 (1.08-1.21) | **0.003** |
| Adenosquamous | 1.20 (1.04-1.37) | **0.010** | 1.16 (1.06-1.28) | **0.001** |
| **Grade** |  |  |  |  |
| G1 | 0.65 (0.54-0.78) | **<0.001** | 0.73 (0.65-0.81) | **<0.001** |
| G2 | 0.84 (0.77-0.91) | **<0.001** | 0.83 (0.79-0.88) | **<0.001** |
| G3 | Ref | - | Ref | - |
| Unknown | 0.78 (0.72-0.85) | **<0.001** | 0.83 (0.79-0.88) | **<0.001** |
| **Surgery** |  |  |  |  |
| No surgery | Ref | - | Ref | - |
| Hysterectomy ^*^ | 0.40 (0.37-0.45) | **<0.001** | 0.39 (0.37-0.42) | **<0.001** |
| Exenteration ^†^ | 0.59 (0.43-0.81) | **0.001** | 0.63 (0.51-0.79) | **0.014** |
| **Radiotherapy** |  |  |  |  |
| No | Ref | - | Ref | - |
| Yes | 0.63 (0.57-0.69) | **<0.001** | 0.63 (0.60-0.67) | **<0.001** |
| **Chemotherapy** |  |  |  |  |
| No/Unknown | Ref | - | Ref | - |
| Yes | 0.80 (0.74-0.86) | **<0.001** | 0.63 (0.60-0.66) | **<0.001** |
| Cox proportional hazard regression models for multivariable analysis. All listed covariates are entered in the final model except for marital status (1988-2003) and year of diagnosis (1988-2003 and 1988-2016). Significant *P* values are in bold form. ^*^ Hysterectomy includes total, modified radical or radical hysterectomy with or without removal of tubes and ovaries. ^†^ Exenteration includes anterior, posterior, total, or extended pelvic exenteration. Abbreviations: CI, confidence interval; DLN+, positive distant lymph nodes; HR, hazard ratio; Ref, reference. | | | | |

| **Supplementary Table 4. Univariable and multivariable analysis for cause-specific survival of patients with T1-T2a and distant lymph node metastasis** | | | | |
| --- | --- | --- | --- | --- |
|  | **Univariable** | | **Multivariable** | |
| **Characteristic** | **HR (90%CI)** | ***P*** | **HR (95%CI)** | ***P*** |
| **Age (years)** |  |  |  |  |
| <40 | Ref | - | - | - |
| 40-49 | 0.92 (0.72-1.19) | 0.599 | - | - |
| 50-59 | 0.92 (0.70-1.21) | 0.626 | - | - |
| 60-69 | 1.22 (0.90-1.66) | 0.278 | - | - |
| ≥70 | 1.18 (0.81-1.71) | 0.462 | - | - |
| **Race** |  |  |  |  |
| White | Ref | - | - | - |
| Black | 1.56 (1.22-2.01) | **0.003** | - | - |
| Others | 1.01 (0.75-1.36) | 0.95 | - | - |
| **Marital Status** |  |  |  |  |
| Single | 1.07 (0.86-1.35) | 0.607 | - | - |
| Married | Ref | - | - | - |
| Divorced | 1.23 (0.93-1.62) | 0.215 | - | - |
| Separated | 1.62 (0.96-2.71) | 0.127 | - | - |
| Widowed | 1.67 (1.18-2.35) | **0.015** | - | - |
| Unknown | 0.92 (0.56-1.51) | 0.779 | - | - |
| **Year of diagnosis** |  |  | - | - |
| 1988-1990 | 1.52 (0.93-2.48) | 0.163 |  |  |
| 1991-1993 | 1.25 (0.78-1.99) | 0.433 |  |  |
| 1994-1996 | 1.16 (0.75-1.79) | 0.587 |  |  |
| 1997-1999 | 1.10 (0.75-1.61) | 0.676 | - | - |
| 2000-2003 | Ref | - | - | - |
| 2004-2006 | 1.19 (0.85-1.66) | 0.395 | - | - |
| 2007-2009 | 1.13 (0.83-1.55) | 0.520 | - | - |
| 2010-2012 | 1.21 (0.89-1.65) | 0.314 | - | - |
| 2013-2016 | 0.90 (0.62-1.31) | 0.657 | - | - |
| **Tumor size** |  |  |  |  |
| ≤4cm | Ref | - | Ref | - |
| >4cm | 1.73 (1.35-2.21) | **<0.001** | 1.45 (1.07-1.96) | **0.016** |
| Unknown | 2.25 (1.76-2.88) | **<0.001** | 1.85 (1.36-2.51) | **<0.001** |
| **T stage** |  |  |  |  |
| T1a | 0.45 (0.20-1.03) | 0.115 | - | - |
| T1b | Ref | - | - | - |
| T1, NOS | 1.82 (1.38-2.41) | **<0.001** | - | - |
| T2a | 1.56 (1.27-1.92) | **<0.001** | - | - |
| **N stage** |  |  |  |  |
| N0 | Ref | - | - | - |
| N1 | 1.08 (0.81-1.44) | 0.653 | - | - |
| NX | 1.72 (1.20-2.47) | **0.014** | - | - |
| **Histology** |  |  |  |  |
| Squamous | Ref | - | - | - |
| Adenocarcinoma | 0.83 (0.65-1.08) | 0.244 | - | - |
| Adenosquamous | 0.94 (0.66-1.34) | 0.770 | - | - |
| **Grade** |  |  |  |  |
| G1 | 0.93 (0.57-1.53) | 0.818 | - | - |
| G2 | 0.82 (0.65-1.04) | 0.162 | - | - |
| G3 | Ref | - | - | - |
| Unknown | 1.23 (0.98-1.53) | 0.137 | - | - |
| **Radiation and surgery ^*^** |  |  |  |  |
| No radiation or surgery | 1.35 (0.94-1.93) | 0.173 | 1.24 (0.80-1.93) | 0.340 |
| Surgery only | 0.89 (0.58-1.39) | 0.665 | 1.05 (0.60-1.84) | 0.862 |
| Combination RT ^†^ | Ref | - | Ref | - |
| EBRT | 1.51 (1.19-1.92) | **0.005** | 1.41 (1.06-1.88) | **0.019** |
| Hysterectomy + combination RT | 0.46 (0.28-0.74) | **0.008** | 0.58 (0.32-1.07) | 0.079 |
| Hysterectomy + EBRT | 0.56 (0.40-0.79) | **0.005** | 0.71 (0.46-1.10) | 0.128 |
| Other regimens ^‡^ | 1.14 (0.82-1.59) | 0.509 | 1.24 (0.83-1.84) | 0.297 |
| **Pelvic lymphadenectomy** |  |  |  |  |
| No/Unknown | Ref | - | Ref | - |
| Yes | 0.56 (0.46-0.67) | **<0.001** | 0.77 (0.59-0.99) | **0.040** |
| **Chemotherapy** |  |  |  |  |
| No/Unknown | Ref | - | Ref | - |
| Yes | 0.95 (0.77-1.18) | 0.720 | 0.92 (0.70-1.21) | 0.538 |
| Cox proportional hazard regression models for univariable and multivariable analysis. Significant *P* values are in bold form. Race, marital status, T stage, and N stage do not enter the multivariable Cox regression model, while chemotherapy was forced into the model. ^*^ Surgery includes hysterectomy and exenteration. Hysterectomy includes total, modified radical or radical hysterectomy with or without removal of tubes and ovaries. Exenteration includes anterior, posterior, total, or extended pelvic exenteration. ^†^ Combination of EBRT and brachytherapy. ^‡^ Other regimens include brachytherapy, radiation (not otherwise specified, NOS), brachytherapy or radiation (not otherwise specified, NOS) after hysterectomy or exenteration, prior ± post-surgery radiotherapy and intraoperative radiotherapy. Abbreviations: CI, confidence interval; EBRT, external beam radiotherapy; HR, hazard ratio; NOS, not otherwise specified; Ref, reference; RT, radiotherapy. | | | | |

| **Supplementary Table 5. Univariable and multivariable analysis for cause-specific survival of patients with T2b-T3 and distant lymph node metastasis** | | | | |
| --- | --- | --- | --- | --- |
|  | **Univariable** | | **Multivariable** | |
| **Characteristic** | **HR (90%CI)** | ***P*** | **HR (95%CI)** | ***P*** |
| **Age (years)** |  |  |  |  |
| <40 | Ref | - | Ref | - |
| 40-49 | 1.04 (0.87-1.25) | 0.697 | 1.01 (0.81-1.26) | 0.955 |
| 50-59 | 0.97 (0.82-1.16) | 0.807 | 0.79 (0.63-0.99) | **0.039** |
| 60-69 | 0.99 (0.82-1.21) | 0.945 | 0.77 (0.60-0.99) | **0.041** |
| ≥70 | 1.72 (1.40-2.11) | **<0.001** | 1.22 (0.91-1.63) | 0.192 |
| **Race** |  |  |  |  |
| White | Ref | - | Ref | - |
| Black | 1.44 (1.23-1.69) | **<0.001** | 1.39 (1.14-1.69) | **0.001** |
| Others | 0.99 (0.82-1.19) | 0.923 | 0.97 (0.78-1.22) | 0.811 |
| **Marital Status** |  |  |  |  |
| Single | 1.00 (0.86-1.15) | 0.975 | 0.90 (0.75-1.08) | 0.240 |
| Married | Ref | - | Ref | - |
| Divorced | 1.22 (1.02-1.46) | **0.067** | 1.29 (1.04-1.61) | **0.022** |
| Separated | 1.04 (0.68-1.58) | 0.883 | 1.20 (0.72-2.00) | 0.479 |
| Widowed | 0.97 (0.80-1.17) | 0.769 | 0.79 (0.62-1.02) | 0.075 |
| Unknown | 1.30 (0.96-1.74) | 0.152 | 1.15 (0.80-1.65) | 0.445 |
| **Year of diagnosis** |  |  |  |  |
| 1988-1990 | 1.86 (1.32-2.67) | **0.003** | - | - |
| 1991-1993 | 1.33 (0.97-1.82) | 0.132 |  |  |
| 1994-1996 | 1.82 (1.41-2.35) | **<0.001** |  |  |
| 1997-1999 | 1.47 (1.12-1.93) | **0.020** |  |  |
| 2000-2003 | Ref | - | - | - |
| 2004-2006 | 1.29 (1.05-1.60) | **0.044** | - | - |
| 2007-2009 | 1.49 (1.21-1.83) | **0.002** | - | - |
| 2010-2012 | 1.41 (1.15-1.72) | **0.005** | - | - |
| 2013-2016 | 1.32 (1.07-1.63) | **0.031** | - | - |
| **Tumor size** |  |  |  |  |
| ≤4cm | Ref | - | - | - |
| >4cm | 1.29 (1.06-1.57) | **0.032** | - | - |
| Unknown | 1.65 (1.35-2.02) | **<0.001** | - | - |
| **T stage** |  |  |  |  |
| T2b | Ref | - | Ref | - |
| T3a | 1.74 (1.44-2.10) | **<0.001** | 1.57 (1.25-1.98) | **<0.001** |
| T3b | 1.74 (1.52-1.98) | **<0.001** | 1.53 (1.30-1.81) | **<0.001** |
| T3, NOS | 1.80 (1.29-2.51) | **0.004** | 1.86 (1.23-2.81) | **0.003** |
| **N stage** |  |  |  |  |
| N0 | Ref | - | - | - |
| N1 | 0.99 (0.82-1.20) | 0.917 | - | - |
| NX | 1.24 (1.00-1.53) | **0.096** | - | - |
| **Histology** |  |  |  |  |
| Squamous | Ref | - | Ref | - |
| Adenocarcinoma | 1.32 (1.10-1.59) | **0.012** | 1.36 (1.08-1.70) | **0.008** |
| Adenosquamous | 1.20 (0.94-1.53) | 0.230 | 1.24 (0.92-1.67) | 0.163 |
| **Grade** |  |  |  |  |
| G1 | 0.98 (0.73-1.30) | 0.891 | - | - |
| G2 | 0.93 (0.81-1.08) | 0.426 | - | - |
| G3 | Ref | - | - | - |
| Unknown | 0.87 (0.75-1.01) | 0.119 | - | - |
| **Radiation and surgery ^*^** |  |  |  |  |
| No radiation or surgery | 3.44 (2.84-4.15) | **<0.001** | 2.71 (2.14-3.44) | **<0.001** |
| Surgery only | 0.92 (0.60-1.40) | 0.733 | 0.87 (0.51-1.48) | 0.606 |
| Combination RT ^†^ | Ref | - | Ref | - |
| EBRT | 1.91 (1.65-2.20) | **<0.001** | 1.74 (1.46-2.08) | **<0.001** |
| Hysterectomy + combination RT | 0.55 (0.35-0.86) | **0.028** | 0.69 (0.40-1.20) | 0.191 |
| Hysterectomy + EBRT | 1.16 (0.88-1.52) | 0.382 | 1.29 (0.92-1.81) | 0.145 |
| Other regimens ^‡^ | 1.22 (0.96-1.56) | 0.177 | 1.26 (0.94-1.70) | 0.123 |
| **Pelvic lymphadenectomy** |  |  |  |  |
| No/Unknown | Ref | - | Ref | - |
| Yes | 0.65 (0.57-0.74) | **<0.001** | 0.83 (0.70-0.98) | **0.030** |
| **Chemotherapy** |  |  |  |  |
| No/Unknown | Ref | - | Ref | - |
| Yes | 0.51 (0.45-0.58) | **<0.001** | 0.54 (0.46-0.64) | **<0.001** |
| Cox proportional hazard regression models for univariable and multivariable analysis. Significant *P* values are in bold form. Year of diagnosis, tumor size, and N stage do not enter the multivariable Cox regression model. ^*^ Surgery includes hysterectomy and exenteration. Hysterectomy includes total, modified radical or radical hysterectomy with or without removal of tubes and ovaries. Exenteration includes anterior, posterior, total, or extended pelvic exenteration. ^†^ Combination of EBRT and brachytherapy. ^‡^ Other regimens include brachytherapy, radiation (not otherwise specified, NOS), brachytherapy or radiation (not otherwise specified, NOS) after hysterectomy or exenteration, prior ± post-surgery radiotherapy, and intraoperative radiotherapy. Abbreviations: CI, confidence interval; EBRT, external beam radiotherapy; HR, hazard ratio; NOS, not otherwise specified; Ref, reference; RT, radiotherapy. | | | | |

| **Supplementary Table 6. Subgroup analysis of the association between chemotherapy and cause-specific survival of patients with T1-T2a and distant lymph node metastasis.** | | | | | | |
| --- | --- | --- | --- | --- | --- | --- |
|  | **Before 1999** |  |  | **After 2000** |  |  |
| **Characteristic** | **HR (95%CI)** | ***P*** | ***P* for Interaction** | **HR (95%CI)** | ***P*** | ***P* for Interaction** |
| **Overall** | 1.25 (0.79-1.99) | 0.349 |  | 0.83 (0.59-1.17) | 0.287 |  |
| **Age** |  |  | 0.889 |  |  | 0.881 |
| <60Y | 1.17 (0.67-2.04) | 0.579 |  | 0.83 (0.55-1.26) | 0.377 |  |
| ≥60Y | 1.57 (0.64-3.88) | 0.328 |  | 0.70 (0.34-1.43) | 0.328 |  |
| **Histology** |  |  | 0.310 |  |  | 0.711 |
| Squamous | 1.05 (0.63-1.74) | 0.856 |  | 0.78 (0.52-1.15) | 0.202 |  |
| Non-Squamous | 3.73 (0.97-14.34) | 0.055 |  | 1.33 (0.65-2.74) | 0.438 |  |
| **T stage** |  |  | 0.995 |  |  | 0.629 |
| T1 | 1.22 (0.67-2.22) | 0.517 |  | 0.82 (0.56-1.22) | 0.330 |  |
| T2a | 1.04 (0.51-2.10) | 0.920 |  | 0.73 (0.36-1.46) | 0.370 |  |
| **Tumor Size** |  |  | 0.691 |  |  | 0.606 |
| <4cm | 1.38 (0.48-3.94) | 0.546 |  | 1.11 (0.50-2.47) | 0.803 |  |
| ≥4cm | 1.15 (0.49-2.73) | 0.749 |  | 0.70 (0.42-1.18) | 0.178 |  |
| Unknown | 1.76 (0.89-3.47) | 0.106 |  | 0.97 (0.53-1.75) | 0.907 |  |
| **Pelvic lymphadenectomy** |  |  | 0.778 |  |  | 0.979 |
| No/Unknown | 1.26 (0.58-2.78) | 0.561 |  | 0.86 (0.54-1.39) | 0.543 |  |
| Yes | 1.13 (0.65-1.95) | 0.665 |  | 0.91 (0.53-1.56) | 0.741 |  |
| **Radiation and surgery** |  |  | 0.722 |  |  | **0.010** |
| Radiation ^*^ | 1.30 (0.71-2.39) | 0.395 |  | 0.54 (0.33-0.90) | **0.017** |  |
| Hysterectomy + PORT ^†^ | 0.82 (0.33-2.05) | 0.678 |  | 0.87 (0.26-2.95) | 0.819 |  |
| Others | 6.10 (1.39-26.78) | 0.016 |  | 1.45 (0.85-2.50) | 0.175 |  |
| Significant *P* values are in bold form. ^*^ Radiation includes combination radiotherapy and external beam radiotherapy. ^†^ Hysterectomy with postoperative combination radiotherapy or postoperative external beam radiotherapy. Abbreviations: CI, confidence interval; HR, multivariable adjusted hazard ratio associated with chemotherapy (no chemotherapy or unknown chemotherapy performance is the reference [HR, 1.0]); PORT, postoperative radiotherapy. | | | | | | |

| **Supplementary Table 7. Demographics and clinical characteristics of patients receiving combination radiotherapy and external beam radiotherapy** | | | | | |
| --- | --- | --- | --- | --- | --- |
|  | **Baseline Characteristics** | | | **Logistic Regression** | |
| **Characteristic** | **Combination RT**  **(n=559)** | **EBRT**  **(n=653)** | ***P* value ^*^** | **OR ^†^**  **(95% CI)** | ***P* value** |
| **Age (years)** |  |  | **<0.001** |  |  |
| <40 | 127 (22.7%) | 104 (15.9%) |  | Ref | - |
| 40-49 | 168 (30.1%) | 156 (23.9%) |  | 0.87 (0.61-1.24) | 0.443 |
| 50-59 | 148 (26.5%) | 166 (25.4%) |  | 0.84 (0.58-1.21) | 0.339 |
| 60-69 | 74 (13.2%) | 122 (18.7%) |  | 0.52 (0.34-0.79) | **0.002** |
| ≥70 | 42 (7.5%) | 105 (16.1%) |  | 0.43 (0.26-0.69) | **0.001** |
| **Race** |  |  | **0.003** |  |  |
| White | 415 (74.2%) | 465 (71.2%) |  | Ref | - |
| Black | 72 (12.9%) | 127 (19.4%) |  | 0.70 (0.50-0.99) | **0.042** |
| Others | 72 (12.9%) | 61 (9.3%) |  | 1.34 (0.89-1.99) | 0.159 |
| **Marital Status** |  |  | **0.006** |  |  |
| Single | 142 (25.4%) | 207 (31.7%) |  | - | - |
| Married | 244 (43.6%) | 238 (36.4%) |  | - | - |
| Divorced | 80 (14.3%) | 75 (11.5%) |  | - | - |
| Separated | 15 (2.7%) | 14 (2.1%) |  | - | - |
| Widowed | 55 (9.8%) | 96 (14.7%) |  | - | - |
| Unknown | 23 (4.1%) | 23 (3.5%) |  | - | - |
| **Year of diagnosis** |  |  | **<0.001** |  |  |
| 1988-1990 | 19 (3.4%) | 15 (2.3%) |  | 1.93 (085-4.37) | 0.117 |
| 1991-1993 | 28 (5.0%) | 20 (3.1%) |  | 1.33 (0.68-2.63) | 0.407 |
| 1994-1996 | 52 (9.3%) | 18 (2.8%) |  | 3.46 (1.78-6.75) | **<0.001** |
| 1997-1999 | 42 (7.5%) | 26 (4.0%) |  | 1.85 (0.99-3.43) | 0.052 |
| 2000-2003 | 112 (20.0%) | 91 (13.9%) |  | Ref | - |
| 2004-2006 | 53 (9.5%) | 94 (14.4%) |  | 0.44 (0.28-0.70) | **0.001** |
| 2007-2009 | 61 (10.9%) | 113 (17.3%) |  | 0.46 (0.30-0.72) | **0.001** |
| 2010-2012 | 73 (13.1%) | 125 (19.1%) |  | 0.49 (0.31-0.75) | **0.001** |
| 2013-2016 | 119 (21.3%) | 151 (23.1%) |  | 0.69 (0.46-1.04) | 0.075 |
| **Histology** |  |  | 0.161 |  |  |
| Squamous | 484 (86.6%) | 540 (82.7%) |  | - | - |
| Adenocarcinoma | 50 (8.9%) | 79 (12.1%) |  | - | - |
| Adenosquamous | 25 (4.5%) | 34 (5.2%) |  | - | - |
| **Grade** |  |  | 0.457 |  |  |
| G1 | 24 (4.3%) | 23 (3.5%) |  | - | - |
| G2 | 156 (27.9%) | 165 (25.3%) |  | - | - |
| G3 | 232 (41.5%) | 270 (41.3%) |  | - | - |
| Unknown | 147 (26.3%) | 195 (29.9%) |  | - | - |
| **Tumor Size** |  |  | **0.002** |  |  |
| ≤4cm | 90 (16.1%) | 70 (10.7%) |  | Ref | - |
| >4cm | 289 (51.7%) | 318 (48.7%) |  | 0.75 (0.51-1.11) | 0.149 |
| Unknown | 180 (32.2%) | 265 (40.6%) |  | 0.57 (0.38-0.86) | **0.007** |
| **T stage** |  |  | **<0.001** |  |  |
| T1 | 97 (17.4%) | 121 (18.5%) |  | Ref | - |
| T2 | 220 (39.4%) | 171 (26.2%) |  | 1.73 (1.21-2.49) | **0.003** |
| T3 | 242 (43.3%) | 361 (55.3%) |  | 1.04 (0.73-1.48) | 0.818 |
| **N stage** |  |  | **0.006** |  |  |
| N0 | 53 (9.5%) | 94 (14.4%) |  | - | - |
| N1 | 366 (65.5%) | 431 (66.0%) |  | - | - |
| NX | 140 (25.0%) | 128 (19.6%) |  | - | - |
| **Pelvic lymphadenectomy** | |  | **<0.001** |  |  |
| No/Unknown | 359 (64.2%) | 511 (78.3%) |  | Ref | - |
| Yes | 200 (35.8%) | 142 (21.7%) |  | 1.35 (1.01-1.81) | **0.041** |
| **Chemotherapy** |  |  | **0.003** |  |  |
| No/Unknown | 80 (14.3%) | 137 (21.0%) |  | Ref | - |
| Yes | 479 (85.7%) | 516 (79.0%) |  | 2.20 (1.50-3.24) | **<0.001** |
| Significant *P* values are in bold form. Combination RT: combination of EBRT and brachytherapy. ^*^ Univariable analysis with chi-square test for *P* values. ^†^ ORs reflect the multivariable adjusted odds ratio of receiving combination RT. Marital status, grade, histology, and N stage did not enter the multivariable logistic regression model. Abbreviations: CI, confidence interval; EBRT, external beam radiotherapy; NOS, not otherwise specified; OR, odds ratio; RT, radiotherapy. | | | | | |

| **Supplementary Table 8. Demographics and clinical characteristics of patients receiving combination radiotherapy and external beam radiotherapy for the propensity–score matched analysis** | | | | |
| --- | --- | --- | --- | --- |
|  |  | **Combination RT** | **EBRT** | ***P* value** |
| **Characteristic** | **N=838** | **n=419** | **n=419** |  |
| **Age (years)** |  |  |  | 0.637 |
| <40 | 164 (19.6%) | 87 (20.8%) | 77 (18.4%) |  |
| 40-49 | 247 (29.5%) | 117 (27.9%) | 130 (31.0%) |  |
| 50-59 | 222 (26.5%) | 113 (27.0%) | 109 (26.0%) |  |
| 60-69 | 123 (14.7%) | 65 (15.5%) | 58 (13.8%) |  |
| ≥70 | 82 (9.8%) | 37 (8.8%) | 45 (10.7%) |  |
| **Race** |  |  |  | 0.509 |
| White | 611 (72.9%) | 300 (71.6%) | 311 (74.2%) |  |
| Black | 122 (14.6%) | 61 (14.6%) | 61 (14.6%) |  |
| Others | 105 (12.5%) | 58 (13.8%) | 47 (11.2%) |  |
| **Marital Status** |  |  |  | 0.503 |
| Single | 240 (28.6%) | 110 (26.3%) | 130 (31.0%) |  |
| Married | 344 (41.1%) | 171 (40.8%) | 173 (41.3%) |  |
| Divorced | 119 (14.2%) | 68 (16.2%) | 51 (12.2%) |  |
| Separated | 22 (2.6%) | 11 (2.6%) | 11 (2.6%) |  |
| Widowed | 86 (10.3%) | 45 (10.7%) | 41 (9.8%) |  |
| Unknown | 27 (3.2%) | 14 (3.3%) | 13 (3.1%) |  |
| **Year of diagnosis** |  |  |  | 0.794 |
| 1988-1990 | 25 (3.0%) | 14 (3.3%) | 11 (2.6%) |  |
| 1991-1993 | 36 (4.3%) | 18 (4.3%) | 18 (4.3%) |  |
| 1994-1996 | 40 (4.8%) | 23 (5.5%) | 17 (4.1%) |  |
| 1997-1999 | 48 (5.7%) | 29 (6.9%) | 19 (4.5%) |  |
| 2000-2003 | 140 (16.7%) | 66 (15.8%) | 74 (17.4%) |  |
| 2004-2006 | 103 (12.3%) | 51 (12.2%) | 52 (12.4%) |  |
| 2007-2009 | 121 (14.4%) | 56 (13.4%) | 65 (15.5%) |  |
| 2010-2012 | 130 (15.5%) | 63 (15.0%) | 67 (16.0%) |  |
| 2013-2016 | 195 (23.3%) | 99 (23.6%) | 96 (22.9%) |  |
| **Histology** |  |  |  | 0.237 |
| Squamous | 718 (85.7%) | 365 (87.1%) | 353 (84.2%) |  |
| Adenocarcinoma | 82 (9.8%) | 40 (9.5%) | 42 (10.0%) |  |
| Adenosquamous | 38 (4.5%) | 14 (3.3%) | 24 (5.7%) |  |
| **Grade** |  |  |  | 0.277 |
| G1 | 34 (4.1%) | 18 (4.3%) | 16 (3.8%) |  |
| G2 | 228 (27.2%) | 125 (29.8%) | 103 (24.6%) |  |
| G3 | 343 (40.9%) | 169 (40.3%) | 174 (41.5%) |  |
| Unknown | 233 (27.8%) | 107 (25.5%) | 126 (30.1%) |  |
| **Tumor Size** |  |  |  | 0.962 |
| ≤4cm | 111 (13.2%) | 55 (13.1%) | 56 (13.4%) |  |
| >4cm | 438 (52.3%) | 221 (52.7%) | 217 (51.8%) |  |
| Unknown | 289 (34.5%) | 143 (34.1%) | 146 (34.8%) |  |
| **T stage** |  |  |  | 1.000 |
| T1 | 126 (15.0%) | 63 (15.0%) | 63 (15.0%) |  |
| T2 | 306 (36.5%) | 153 (36.5%) | 153 (36.5%) |  |
| T3 | 406 (48.4%) | 203 (48.4%) | 203 (48.4%) |  |
| **N stage** |  |  |  | 0.636 |
| N0 | 100 (11.9%) | 47 (11.2%) | 53 (12.6%) |  |
| N1 | 544 (64.9%) | 270 (64.4%) | 274 (65.4%) |  |
| NX | 194 (23.2%) | 102 (24.3%) | 92 (22.0%) |  |
| **Pelvic lymphadenectomy** | |  |  | 0.647 |
| No/Unknown | 598 (71.4%) | 302 (72.1%) | 296 (70.6%) |  |
| Yes | 240 (28.6%) | 117 (27.9%) | 123 (29.4%) |  |
| **Chemotherapy** |  |  |  | 0.198 |
| No/Unknown | 119 (14.2%) | 66 (15.8%) | 53 (12.6%) |  |
| Yes | 719 (85.8%) | 353 (84.2%) | 366 (87.4%) |  |
| Univariable analysis with chi-square test for *P* values. Combination RT: combination of EBRT and brachytherapy. Covariates used to generate propensity scores include age, race, year of diagnosis, tumor size, T stage, pelvic lymphadenectomy, and chemotherapy performance. **After matching, the standardized difference of all predictors between the radiotherapy groups <0.10.** Abbreviations: EBRT, external beam radiotherapy; RT, radiotherapy. | | | | |

| **Supplementary Table 9. Results of previous studies of chemoradiotherapy of patients with metastatic cervical cancer.** | | | | | | | | |
| --- | --- | --- | --- | --- | --- | --- | --- | --- |
|  | **N** | **Primary Tumor**  **(n)** | **Metastatic Sites**  **(n)** | **Pelvis Radiation** | | **EBRT to Metastatic Sites**  **(dose, n)** | **Chemotherapy**  **(n)** | **Survival** |
|  |  |  |  | **Treatment (n)** | **Dose** |  |  |  |
| Tran (2003) 1998-2002  USA (19) | 11 | T1, 2;  T2, 2;  T3, 7. | PALN, 11;  SCLN, 11 | EBRT + BT, 10;  EBRT alone, 1 | EBRT + HDR ICR 60.8Gy  EBRT + LDR ICR 81.7-86.5Gy | PALN, 45Gy, 7;  SCLN, 45Gy, 7. | Concurrent, 11 | mOS 7.5m |
| Kim (2010)  1997-2008  Korea (20) | 13 | NA | SCLN, 13 | EBRT, 11;  BT, NA ^*^ | EBRT + ICR 81-91Gy | PALN, 50-70Gy；  Mediastinal LN, 45-56Gy；  SCLN，59-72Gy | Concurrent, 9;  CT alone, 2;  No CT, 2 | 2-y OS = 75% |
| Kim (2012)  1998-2010  Korea (7) | 25 | NA | PALN, 25;  SCLN, 25 | EBRT + BT, 22;  EBRT alone, 3 | EBRT 50.4Gy+HDR ICR 30Gy | PALN, 59.4Gy;  SCLN, 59.4Gy | Concurrent, 25 | mOS, 32m;  3-y OS = 49% |
| Lee (2012)  2001-2009  Korea (21) | 7 | NA | PALN, 7;  SCLN, 7 | EBRT + BT, 7 | EBRT 55.8Gy + HDR ICR 24Gy | PALN, 54Gy;  SCLN, 66.6Gy | Concurrent, 7 | 5-y OS = 57.1% |
| Kim (2013)  2000-2010  Korea (22) | 24 | NA | DLN beyond abdomen | EBRT + BT, 5;  EBRT alone, 5 | EBRT 50.4Gy + ICR 33.5-35Gy | PALN, 45-61.2 Gy, 7  SCLN, 61.2-70.2 Gy, 3 | CRT, 10:  Systemic CT, 14 | CRT mOS 63.7m;  Systemic CT alone mOS 18.4m |
| Im (2015)  1980-2012  Korea (23) | 35 | NA | DLN beyond abdomen | EBRT + BT, 35; | EBRT 45Gy + HDR ICR 30Gy | DLN, 45Gy, 28 | Concurrent, 25  No CT, 10 | 5-y OS = 46.3% |
| Venigalla (2018)  2004-2013  USA ^†^ (16) | 2838 | T1, 219  T2, 551  T3, 1066  T4, 477  Unknown, 525 | DLN,  distant organs | EBRT>45Gy:  EBRT + BT, 475;  EBRT alone, 594  EBRT≤45Gy，771 | NA | NA | CT, 2838 | Definitive local therapy  2-y OS = 43.1%  Conservative therapy  2-y OS = 21.7% ^‡^ |
| Wang (2018)  2000-2014  USA ^†^ (17) | 3169 | NA | DLN,  distant organs | EBRT + BT, 670;  EBRT alone, 1691 | NA | NA | CRT, 2361;  CT alone, 808 | CT+EBRT+BT 2-y OS = 55%  CT+EBRT 2-y OS = 29%  CT alone 2-y OS = 23% |
| Perkins (2020)  2005-2015  USA (18) | 95 | NA | DLN, distant organs | EBRT, 34 | NA | NA | CT + EBRT, 34;  CT alone, 61 | CT + EBRT mOS 41.6m;  CT alone mOS 17.6m |
| Present Study  1988-2016  USA | 1212 | T1, 218;  T2, 391;  T3, 603 | DLN | EBRT + BT, 559;  EBRT, 653 | NA | NA | CT, 995  No CT/unknown, 217 | EBRT + BT 5-y CSS = 38.0%  EBRT alone 5-y CSS = 21.7% |
| ^*^ Number of patients who received brachytherapy was not available. ^†^ Population-based study of National Cancer Database. ^‡^ Definitive local therapy = concurrent chemoradiation (dose>45Gy) ± brachytherapy boost or hysterectomy. Conservative therapy = systemic therapy ± palliative EBRT (≤dose 45Gy). Abbreviations: BT, brachytherapy; CRT, chemoradiotherapy; CSS, cause-specific survival; CT, chemotherapy; DLN, distant lymph nodes EBRT, external beam radiotherapy; HDR, high-dose rate; ICR, intracavitary radiation; LDR, low-dose rate; NA, not available; OS, overall survival; PALN, para-aortic lymph nodes; SCLN, supraclavicular lymph nodes. | | | | | | | | |
